# Independent phenotypic plasticity axes define distinct obesity sub-types

**DOI:** 10.1038/s42255-022-00629-2

**Published:** 2022-09-12

**Authors:** Chih-Hsiang Yang, Luca Fagnocchi, Stefanos Apostle, Vanessa Wegert, Salvador Casaní-Galdón, Kathrin Landgraf, Ilaria Panzeri, Erez Dror, Steffen Heyne, Till Wörpel, Darrell P. Chandler, Di Lu, Tao Yang, Elizabeth Gibbons, Rita Guerreiro, Jose Bras, Martin Thomasen, Louise G. Grunnet, Allan A. Vaag, Linn Gillberg, Elin Grundberg, Ana Conesa, Antje Körner, PERMUTE, Timothy Triche, Adelheid Lempradl, Zachary J. DeBruine, Emily Wolfrum, Zachary Madaj, Tim Gruber, Brooke Grimaldi, Andrea Parham, Mitchell J. McDonald, Joseph H. Nadeau, Ildiko Polyak, Carmen Khoo, Christine Lary, Peter D. Gluckman, Neerja Karnani, David Carey, Ruth J. F. Loos, Gabriel Seifert, J. Andrew Pospisilik

**Affiliations:** 1 Van Andel Institute, Grand Rapids, MI USA; 2 Max Planck Institute of Immunobiology and Epigenetics, Freiburg, Germany; 3 BioBam Bioinformatics, Valencia, Spain; 4 Medical Faculty, University of Leipzig, University Hospital for Children & Adolescents, Center for Pediatric Research Leipzig, Leipzig, Germany; 5 Roche Diagnostics Deutschland, Mannheim, Germany; 6 Department of Neurodegenerative Science, Van Andel Institute, Grand Rapids, MI USA; 7 Department of Endocrinology, Rigshospitalet, Copenhagen, Denmark; 8 Steno Diabetes Center Copenhagen, Herlev, Denmark; 9 Department of Biomedical Sciences, University of Copenhagen, Copenhagen, Denmark; 10 Genomic Medicine Center, Children’s Mercy Research Institute, Children’s Mercy Kansas City, MO USA; 11 Institute for Integrative Systems Biology, Spanish National Research Council (CSIC), Paterna, Valencia, Spain; 12 Microbiology and Cell Science Department, University of Florida, Gainesville, FL USA; 13 Helmholtz Institute for Metabolic, Obesity and Vascular Research (HI-MAG) of the Helmholtz Zentrum München at the University of Leipzig and University Hospital Leipzig, Leipzig, Germany; 14 Present Address: Lund University Diabetes Centre, Lund University, Malmö, Sweden; 15 Applied Computing Institute, Grand Valley State University, Grand Rapids, MI, USA; 16 Center for Molecular Medicine, MaineHealth Institute for Research, Scarborough, ME USA; 17 Center for Interdisciplinary Population and Health Research, Maine Health Institute for Research, Portland, ME USA; 18 Liggins Institute, University of Auckland, Auckland, New Zealand; 19 Department of Biochemistry, Yong Loo Lin School of Medicine, National University of Singapore, Singapore, Singapore; 20 Singapore Institute for Clinical Sciences, Agency for Science, Technology and Research, Singapore, Singapore; 21 Bioinformatics Institute, Agency for Science, Technology and Research, Singapore, Singapore; 22 Geisinger Health System, Danville, PA USA; 23 Charles Bronfman Institute for Personalized Medicine, Icahn School of Medicine at Mount Sinai, New York, NY USA; 24 Novo Nordisk Foundation Center for Basic Metabolic Research, Faculty of Health and Medical Science, University of Copenhagen, Copenhagen, Denmark; 25 Department of General and Visceral Surgery, University Medical Center Freiburg, University of Freiburg, Freiburg, Germany

**Keywords:** Epigenetics, Obesity, Genetics research

## Abstract

Studies in genetically ‘identical’ individuals indicate that as much as 50% of complex trait variation cannot be traced to genetics or to the environment. The mechanisms that generate this ‘unexplained’ phenotypic variation (UPV) remain largely unknown. Here, we identify neuronatin (NNAT) as a conserved factor that buffers against UPV. We find that *Nnat* deficiency in isogenic mice triggers the emergence of a bi-stable polyphenism, where littermates emerge into adulthood either ‘normal’ or ‘overgrown’. Mechanistically, this is mediated by an insulin-dependent overgrowth that arises from histone deacetylase (HDAC)-dependent β-cell hyperproliferation. A multi-dimensional analysis of monozygotic twin discordance reveals the existence of two patterns of human UPV, one of which (Type B) phenocopies the NNAT-buffered polyphenism identified in mice. Specifically, Type-B monozygotic co-twins exhibit coordinated increases in fat and lean mass across the body; decreased *NNAT* expression; increased HDAC-responsive gene signatures; and clinical outcomes linked to insulinemia. Critically, the Type-B UPV signature stratifies both childhood and adult cohorts into four metabolic states, including two phenotypically and molecularly distinct types of obesity.

## Abstract (web summary)

Yang et al. show that neuronatin (NNAT) can explain part of the phenotypic variation of complex traits, independently of genetics or the environment. Such NNAT-dependent variations can stratify human cohorts into four metabolic sub-types, including two distinct types of obesity.

## Main

Biomedical research is driven by a 100-year-old dogma that phenotype results from the additive effects of genes and environment^1,2^. Since the 1920s, a persistent and compelling body of evidence has argued for the existence of an additional dimension of phenotypic variation not explained by genetics or the environment^3^. Klaus Gärtner’s 30-year effort to standardize rodent models, for instance, aptly demonstrated that continuously inbred animals raised under stringent, standardized conditions continue to exhibit a remarkable degree of UPV^4^. Potential mediators of UPV include residual genetic variation^5^, mosaic genetic variation, gene–gene and gene–environment interactions (non-additive modifier effects), intergenerational and developmental programming and probabilistic mechanisms such as those underpinning organismal polyphenisms and meta-stable epiallele control^6,7^. For precision medicine, our limited understanding of UPV represents a massive source of untapped potential: estimates from trait concordance analyses between co-twins^8^ suggest that UPV is responsible for ~50% of relevant complex trait variation^9–15^.

Deep literature on epigenetics demonstrates the existence of highly conserved, molecular machinery that stabilize transiently plastic transcriptional units into highly stable ON or OFF transcriptional (phenotypic) outputs between isogenic cells and organisms^16,17^. Literature on position-effect variegation for instance, highlights the existence of hundreds of such genomic loci whose expression output is transiently probabilistic in early development and ultimately deterministic (ON or OFF) despite originating in the same tissue of the same individual in the same environment, without change in the underlying DNA sequence. These studies indicate that a fraction of UPV is likely not due to random biological ‘noise’. The existence of alternate but distinct phenotypic sub-states, as opposed to random phenotypic noise, carries profound implications for precision medicine. While not typically interpreted in this fashion, the original work that pioneered the discovery of epigenetic silencing mechanisms in yeast and *Drosophila*, demonstrate a complex regulatory network exists sufficient to underpin organismal UPV^18–20^, at least as they pertain to single reporter loci. While it is now clear that hormones and chromatin pathways can regulate UPV, we know very little about the molecular machinery that buffers against phenotypic variation and confine developmental/phenotypic outcomes to a specific range for any given gene–environment context. Notably, while conceptually related, the regulation of robustness is thought to be distinct from that of phenotypic plasticity^21–23^. For instance, plasticity regulators inherently mediate gene–environment interaction; robustness factors prevent phenotypic variation upon environmental perturbations^22,23^.

One challenge when studying UPV (and phenotypic variability in general) is the large number of experimental animals required to statistically test and validate variance heterogeneity effects (differences in distribution or variance)^24,25^. Experimental designs must factor in (and rule out) confounds such as paternal, maternal and litter-size effects^26–29^. Most biomedical experiments are not designed or powered for such analyses. Using proper design and power, we recently demonstrated that *Trim28* is a robustness factor in mice; *Trim28* buffers against UPV^30^. This work suggested *Nnat* and imprinted gene network 1 (*IGN1*) as potential mediators of *Trim28*-dependent UPV control.

*Nnat* is a paternally expressed imprinted gene that encodes for a transmembrane proteolipid of the endoplasmic reticulum (ER). It was first described as a developmentally regulated gene of the embryonic brain^31,32^, but is also widely expressed and associated with energy homeostasis across tissues^33^. *Nnat* expression is necessary for proper glucose-stimulated insulin secretion in differentiated pancreatic β-cells^34–36^, for adipogenesis and glucose disposal in adipocytes^37–39^, appetite in the hypothalamus^40^ and for energy expenditure and food intake^41,42^. It remains unclear whether any of these functions play a causal role in the emergence of UPV or mammalian polyphenism.

Here, we find that: (1) *Nnat* insufficiency triggers an overgrowth polyphenism (increased fat and lean mass) distinct and independent of *Trim28*-buffered UPV^43^; (2) *Nnat*- and *Trim28*-buffering mechanisms are distinct; (3) *Nnat*-buffered overgrowth is driven by cell-autonomous β-cell hyperplasia and can be abrogated by chemical intervention; and (4) that β-cell hyperplasia depends on HDAC-dependent transcriptional rewiring. Expanding our analysis to humans, we identify at least two different, recurrent patterns of human UPV among monozygotic (MZ) co-twins (Type A and Type B). Of note, Type-B UPV is associated with reduced *NNAT* gene expression and shares similar molecular and metabolic features with the mouse model. Critically, a Type-B UPV gene expression signature stratifies human populations into distinct molecular/metabolic sub-types and separates two types of obesity. The data reported here therefore identify NNAT as a critical regulator of mammalian UPV.

## Results

### *Nnat* buffers an overgrowth polyphenism

**Fig. 1:**
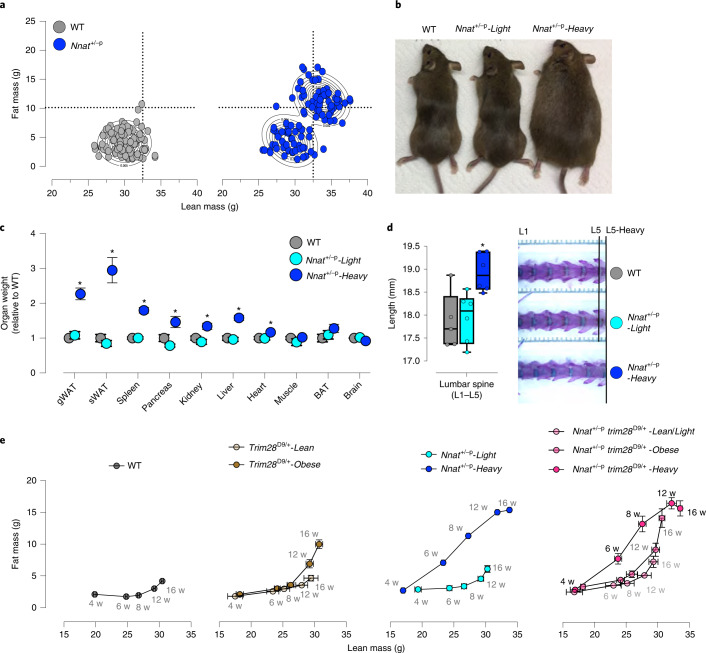
Paternal *Nnat* deletion triggers a bi-stable epigenetic overgrowth in mice. **a**, Body composition shown for 16-week-old F1 male progeny from *Nnat*^+/-p^ × FVBN/J crosses. Contour plots highlighted main clusters identified by Gaussian finite mixture modeling. **b**, Representative picture presented for *Nnat*^+/p^ isogenic morphs and WT littermates. **c**, Organ masses were measured from *Nnat*^+/p^ colony. Each group had at least eight animals. *Adjusted *P* ≤ 0.05, as assessed by one-sided Tukey’s multiple comparisons test, comparing *Nnat*^+/p^-*Heavy* and *Nnat*^+/p^-*Light* littermates. Specifically, gonadal white adipose tissue (gWAT) *P* < 0.0001, subcutaneous white adipose tissue (sWAT) *P* < 0.0001, spleen *P* < 0.0001, pancreas *P* = 0.0019, kidney *P* = 0.0011, liver *P* < 0.0001 and heart *P* < 0.0297. Data are presented as mean ± s.e.m. BAT, brown adipose tissue. **d**, The lumbar spine (L1–L5) length was measured for the *Nnat*^+/p^ colony. Each group had at least five animals. In all box-plots, the lower and upper hinges represent 25th and 75th percentiles. The upper/lower whiskers represent largest/smallest observation less/greater than upper/lower hinge+1.5 × interquartile range (IQR). Central median represents 50% quantile. *Adjusted *P* = 0.015) as assessed by one-sided Tukey’s multiple comparisons test. **e**, Body composition (fat and lean mass) was measured via EchoMRI for each F1 male progeny at 4, 6, 8, 12 and 16 weeks from B6.*Nnat*^+/-p^ × FVB.*Trim28*^D9/+^ crosses. Developmental trajectories according to the phenotypic groups were plotted from 4 to 16 weeks. Each trajectory had at least four animals. Data are presented as mean ± s.e.m. *P ≤ 0.05 by Student’s *t*-test. Source data

**Extended Data Fig. 1:**
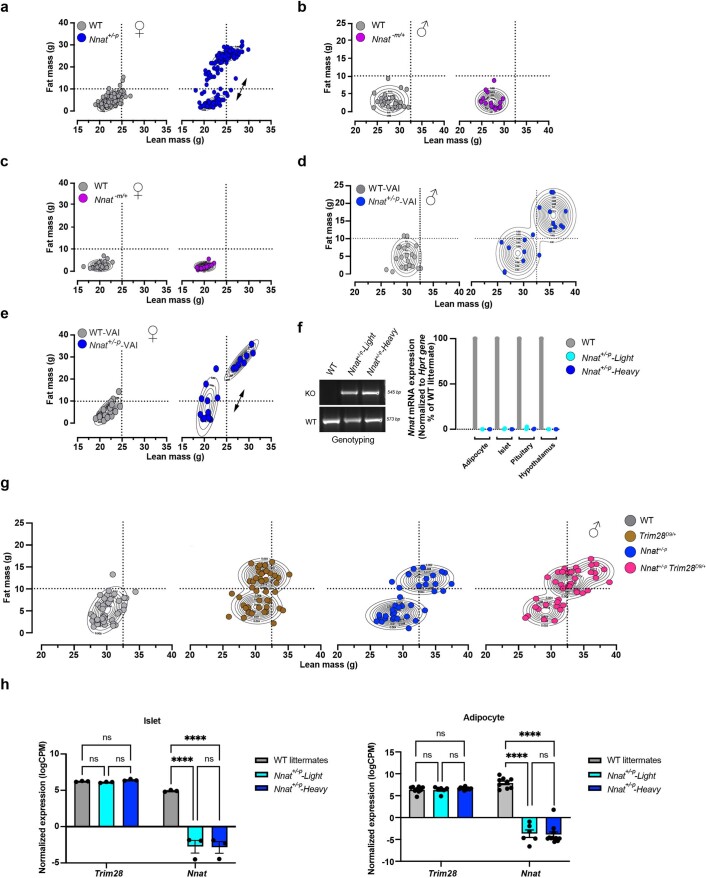
Paternal but not maternal *Nnat* deletion causes bi-stable overgrowth. **a**, The body composition was shown for 16 weeks old of F1 female wild-type and *Nnat*^+^^*/-p*^ animals from *Nnat*^+^^*/-p*^ x FVBN/J crosses. **b**-**c**, The body composition was shown for 16 weeks old F1 male (**b**) and female (**c**) wild-type and *Nnat*^*m/+*^ animals from *Nnat*^*-m/+*^ x FVBN/J crosses. **d**-**e**, The *Nnat*^+^^*/-p*^ male (**d**) and female (**e**) body composition was plotted for the Nnat^+^^*/-p*^ colony in the vivarium, VAI, U.S.A. **f**, Genotyping (DNA) was confirmed in the indicated *Nnat*^+^^*/-p*^ morphs and WT littermate. *Nnat* mRNA expression was confirmed in metabolic tissues (adipocytes, islet, pituitary gland and hypothalamus) from male *Nnat*^+^^*/-p*^ mice. *Nnat* mRNA expression was measured from two independent sets of littermate matched animals. The experiments were repeated independently 3 times with similar results. **g**, Body composition was measured by EchoMRI on 16-week-old wild-type (WT), *Trim28*^*D9/+*^, *Nnat*^+^^*/p*^ and *Nnat*^+^^*/p*^ *Trim28*^*D9/+*^ male progeny from F1 of B6.*Nnat*^+^^*/-p*^ x FVB.*Trim28*^*D9/+*^ crosses. Contour plots highlight main clusters identified by Gaussian finite mixture modeling. **h**, *Trim28* and *Nnat* (as control) gene expression were shown from islet (left) and adipocyte (right) transcriptomes from *Nnat*^+^^*/-p*^ colony. Each group had at least 3 animals. All data were plotted as mean ± SEM, ns (not significant), **** (adjusted *p* < 0.0001) by one-sided Tukey’s multiple comparisons test. Source data

To unequivocally test Nnat’s role as a robustness factor and understand the physiological mechanisms by which it buffers against UPV, we intercrossed highly inbred B6 congenic *Nnat* knockout males (B6.*Nnat*^+/-p^) with wild-type (WT) FVBN/J females, generating large cohorts of *Nnat*-deficient (*Nnat*^+/-p^) and WT littermate matched F1 controls. This breeding scheme maximally restricted inter-individual genetic variation (through isogenicity) while maintaining substantial genome-wide heterozygosity. To minimize litter-size effects (variation attributable to differences in in utero/early-life sufficiency), we used offspring from litters of 9–12 pups and tightly controlled husbandry, environment and housing density. *Nnat*^+/-p^ mice emerged into adulthood in one of two non-overlapping (bi-stable) phenotypic forms: either unremarkable (*Nnat*^+/-p^*-Light*) or overgrown relative to WT and *Nnat*^+/-p^-*Light* animals (*Nnat*^+/-p^*-Heavy*). Overgrowth was characterized by coordinated increases in fat and lean mass (Fig. 1a,b and Extended Data Fig. 1a), which is distinct from the previously reported *Trim28*-buffered polyphenism and from other reports of heterogeneity using the *Nnat*^+/-p^ allele^36,41–43^. *Nnat*^+/-p^*-Heavy* animals were ~50% heavier than both their WT and *Nnat*^+/-p^*-Light* littermates and had increased white adipose tissue, spleen, pancreas, kidney, liver and heart mass (Fig. 1c). Notably, not all tissues were enlarged. Skeletal muscle, brown adipose tissue and brain masses were unchanged or minimally reduced in *Nnat*^+/-p^*-Heavy* animals (Fig. 1c, right). Skeletal morphometry confirmed a larger skeletal frame in the *Nnat*^+/-p^*-Heavy* morphs (Fig. 1d). Given that the *Nnat*^+/-p^-*Light* and *Nnat*^+/-p^*-Heavy* animals are isogenic, raised in highly standardized environments and reproducibly observed within litters and across multiple independent breeding pairs, these data demonstrate that *Nnat* acts to buffer against bi-stable overgrowth potential. Thus, *Nnat* deficiency triggers a polyphenism, characterized by probabilistic overgrowth and obesity.

We validated these findings in several ways. First, deleting the imprinted maternal *Nnat* allele, which generates isogenic *Nnat*-deletion mutants albeit with normal *Nnat* expression, did not trigger overgrowth in the same line (*Nnat*^-m/+^; Extended Data Fig. 1b,c). Second, we tested and observed phenotypic bi-stability in two independent mouse houses and after surviving rederivations independently from cryopreserved embryos and sperm (MPI-IE, Germany; Fig. 1a and Extended Data Fig. 1a; and VAI, USA; Extended Data Fig. 1d,e). Third, we observed bi-stability over dozens of generations, despite exclusively using *Nnat*^+/-p^*-Light* animals as fathers and naive WT C57BL/6J females for continuous backcrossing. *Nnat* deletion is clear in both *Light* and *Heavy* morphs at the DNA and messenger RNA levels (Extended Data Fig. 1f). Thus, *Nnat*^+/-p^-triggered overgrowth is robust across distinct environments and in vitro rederivation protocols. To the best of our knowledge, these data represent an unprecedented demonstration of mammalian polyphenism in a genetic context of substantial genome-wide heterozygosity (F1 hybrids as opposed to congenic lines). They rule out genome-wide homozygosity as a precondition for mammalian polyphenism.

One of the key challenges in deciphering mechanisms that regulate UPV is our limited understanding of the fidelity with which UPV effects are manifest across disparate experimental conditions. We therefore used genetic epistasis to test whether *Trim28* and *Nnat*-buffered polyphenisms are simply context-specific forms of the same process. We crossed FVB.*Trim28*^+/D9^ (maternal) and B6.*Nnat*^+/-p^ (paternal) lines to generate B6/FVB F1 hybrid offspring that were either WT at both loci, mutant only for *Trim28*^+/D9^, mutant only for *Nnat*^+/-p^ or mutant for both alleles in the very same genetic background, parental and in utero contexts. *Trim28*^+/D9^ offspring (WT for *Nnat)* showed bi-stable growth trajectories culminating in a bi-stable obesity, whereas solely their *Nnat*^+/-p^ siblings (WT for *Trim28*) showed distinct early bifurcating overgrowth trajectories (Fig. 1e). These data indicated the *Trim28-* and *Nnat*-induced polyphenisms are at least partially distinct. Notably and in the true test of independence, double-mutant (*Nnat*^+/-p^;*Trim28*^+/D9^) littermates showed tri-stable phenotypic trajectories (Fig. 1e and Extended Data Fig. 1g), where genetically and context-matched animals exhibited a light (WT-like), obese or overgrown phenotype. *Trim28* expression was unchanged among WT, *Nnat*^+/-p^*-Light* and *Nnat*^+/-p^-*Heavy* animals (Extended Data Fig. 1h). The single-mutant analyses also indicate that *Nnat-*mediated buffering is agnostic to the loss of maternal *Trim28;* and that *Trim28-*dependent buffering is agnostic to the loss of paternal *Nnat*^+/^^−^. This data shows unprecedented genetic proof of tri-stable phenotypic potential in mammals and demonstration of independence and additivity of distinct polyphenisms. The data also indicate that the probabilistic ‘obese’ and ‘heavy’ morphs triggered by loss of *Trim28* and *Nnat* (respectively) are distinct and they demonstrate that the mammalian genome has the capacity to canalize three reproducible and discrete developmental trajectories.

### *Nnat* loss triggers bi-stable β-cell hyperplasia

**Extended Data Fig. 2:**
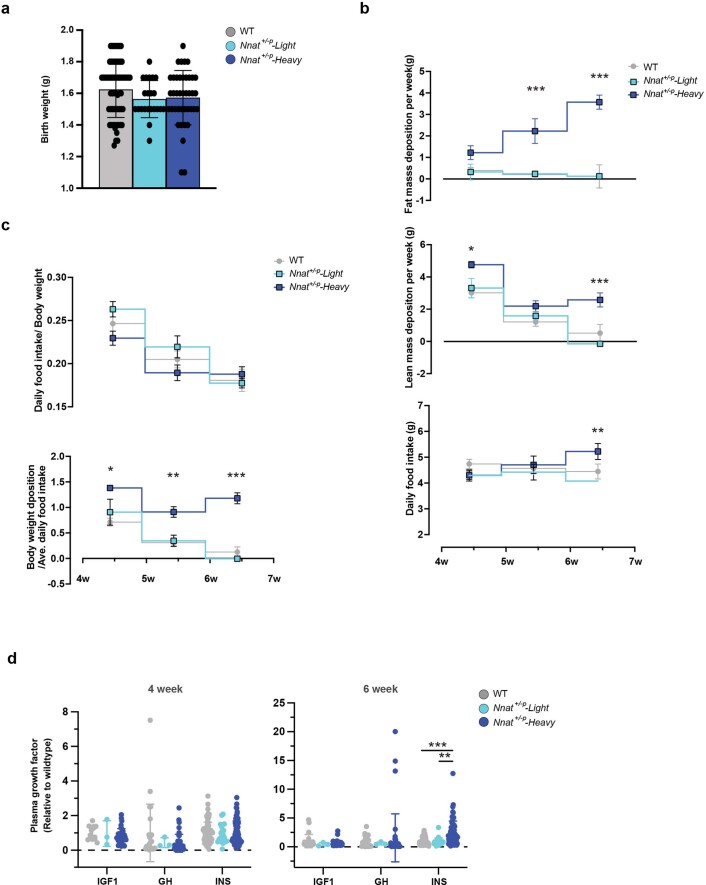
*Nnat*^*+/-p*^-Heavy morphs exhibit accelerated post-weaning growth kinetics associated with hyperinsulinemia. **a**, Birth weight was measured for *Nnat*^+^^*/-p*^ male newborns. Each group had at least 22 animals. Data are presented as mean ± SEM. **b**, Fat (top) and lean (middle) mass deposition between 4 to 7 weeks were plotted for male *Nnat*^+^^*/-p*^ colony. Daily food intake (button) was measured and the average daily food intake were calculated per week between 4 to 7 weeks for male *Nnat*^+^^*/-p*^ colony. All data were plotted as mean ± SEM. Adjusted *p-*values by one-sided Tukey’s multiple comparisons test. Top panel *** *p* < 0.0001; middle panel * *p* = 0.016, *** *p* < 0.0001; lower panel * *p* = 0.0019. **c**, Daily food intake normalized to body weight (top) and body weight deposition normalized to average daily food intake per week (button) between 4 to 7 weeks were plotted for male *Nnat*^+^^*/-p*^ colony. All data were plotted as mean ± SEM. Adjusted *p-*values by one-sided Tukey’s multiple comparisons test. Lower panel * *p* = 0.0104, ** *p* = 0.002, *** *p* < 0.0001. **d**, Circulating growth factors (insulin-like growth factor 1, IGF1; growth hormone, GH; and insulin, INS) were detected in plasma of *Nnat*^+^^*/p*^ males and WT littermates at 4 and 6 weeks of age. Each group had at least 3 animals. All data were plotted as mean ± SEM. Adjusted *p-*values by one-sided Tukey’s multiple comparisons test. ** *p* = 0.0001, *** *p* < 0.0001. Source data

Previous work suggested that *Nnat* deletion causes a stochastic obesity (B6 background; several groups/vivaria^42,43^) and partially penetrant, early-life growth restriction that is alleviated in later life through increased food intake (129S2/Sv background; one group^41^). To capture early growth kinetics of the *Nnat*^+/-p^-*Light* and *Nnat*^+/-p^-*Heavy* animals, we tattooed animals at birth and tracked body composition. *Nnat*^+/-p^-*Light* and *Nnat*^+/-p^-*Heavy* animals exhibited comparable birth weights (Extended Data Fig. 2a), but *Nnat*^+/-p^-*Heavy* morphs initiated the overgrowth developmental trajectory just after 4 weeks of age (Fig. 1e and Extended Data Fig. 2b; top and middle). The phenotypic bifurcation is distinct from that observed in *Trim28* mutant mice that bifurcate (toward lean or obese end states) in early adulthood (8–12 weeks; Fig. 1e). To better understand the origins of phenotypic bifurcation, we measured food intake and body composition changes of the *Nnat*^+/-p^ animals between 4 and 7 weeks of age. Of note, increases in lean and fat mass were measurable several weeks before any detectable hyperphagia, arguing against hyperphagia as a driver of *Nnat*^+/-p^-associated overgrowth and adiposity (Extended Data Fig. 2b,c).

**Fig. 2:**
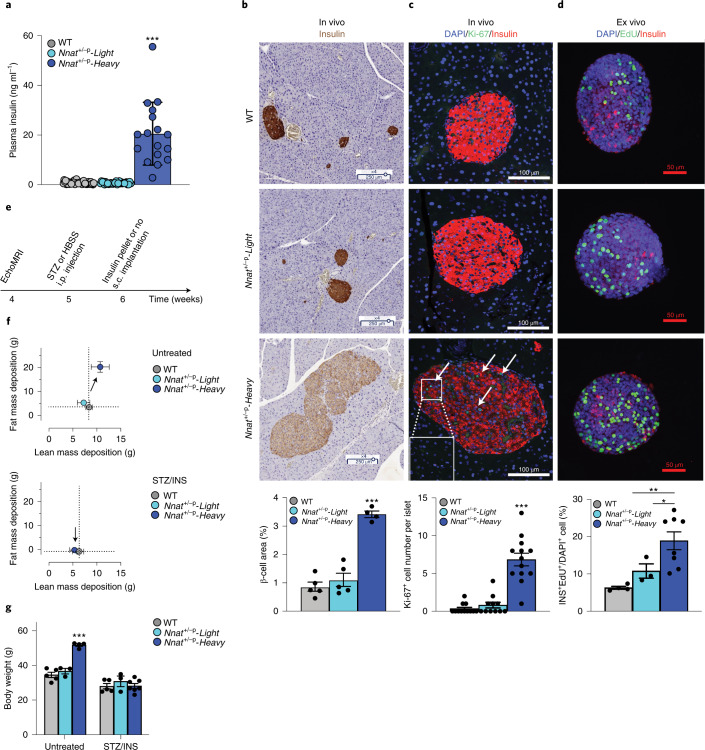
*Nnat*^+/-p^-overgrowth exhibits autonomous β-cell hyperplasia and hyperinsulinemia. **a**, Plasma insulin was measured from 16-week-old male animals fasted for 6 h. Each group had at least 17 animals. ***Adjusted *P* ≤ 0.001, as assessed by one-sided Tukey’s multiple comparisons test. **b**, Insulin-positive β-cells (brown) in *Nnat*^+/-p^ pancreata were detected by immunohistochemistry staining. Scale bar, 250 µm. β-cell area was quantified as percentage of the entire pancreas area. Each group had at least four animals. ***Adjusted *P* ≤ 0.001, as assessed by one-sided Tukey’s multiple comparisons test. **c**, In vivo immunofluorescence was performed for proliferating β-cells (white arrows) in primary islets from 16-week-old animals (red, insulin; blue, DAPI; green, Ki-67). Scale bar, 100 µm. Ki-67^+^ β-cells were quantified and each group had at least 11 islets. ***Adjusted *P* ≤ 0.001, as assessed by one-sided Tukey’s multiple comparisons test. DAPI, 4,6-diamidino-2-phenylindole. **d**, Ex vivo immunofluorescence was performed for proliferating β-cells by EdU-incorporation. Size-matched primary islets from 5–6-week-old mice were cultured for 3 days before the EdU-incorporation assay (red, insulin; blue, DAPI; green, EdU). Scale bar, 50 µm. EdU^+^ proliferating β-cells were quantified and each group had at least three islets. *Two-tailed *P* ≤ 0.05, **two-tailed *P* ≤ 0.01, by Welch’s *t*-test. **e**, STZ (300 mg kg^−1^) was administered at 5 weeks of age when the *Nnat*^+/-p^-Heavy morphs first show signs of accelerated weight gain. An equal number of subcutaneous (s.c.) insulin implants were administered after 5 days and 1 month after the STZ injection to all STZ groups such that relative euglycemia was maintained. i.p., intraperitoneal. **f**, Lean and fat mass gained between 4 and 12 weeks of age for untreated and STZ-treated *Nnat*^+/-p^ littermates. Each group has at least three animals. INS, insulin. **g**, Body weight at termination highlights how *Nnat*^+/-p^-Heavy mice fail to exhibit the overgrowth phenotype on combined STZ/insulin treatment. ***Adjusted *P* = 0.0001, as assessed by one-sided Tukey’s multiple comparisons test. All data are presented as mean ± s.e.m. Source data

Clinically, overgrowth typically results from hyperactive growth hormone (GH)/insulin-like growth factor (IGF) signaling. We found no increase in GH, IGF1 or IGF2 (undetectable) in *Nnat*^+/-p^-*Heavy* mice, suggesting non-canonical overgrowth (Extended Data Fig. 2d). GH was slightly reduced in *Nnat*^+/-p^-*Heavy* mice at 4 weeks, but not significantly. Notably, plasma levels of insulin (a non-canonical activator of IGFR signaling) showed marked increases in *Nnat*^+/-p^-*Heavy* animals simultaneously with overgrowth bifurcation (Extended Data Fig. 2d), reaching an exceptional ~20-fold normal levels by 16 weeks of age (Fig. 2a).

**Extended Data Fig. 3:**
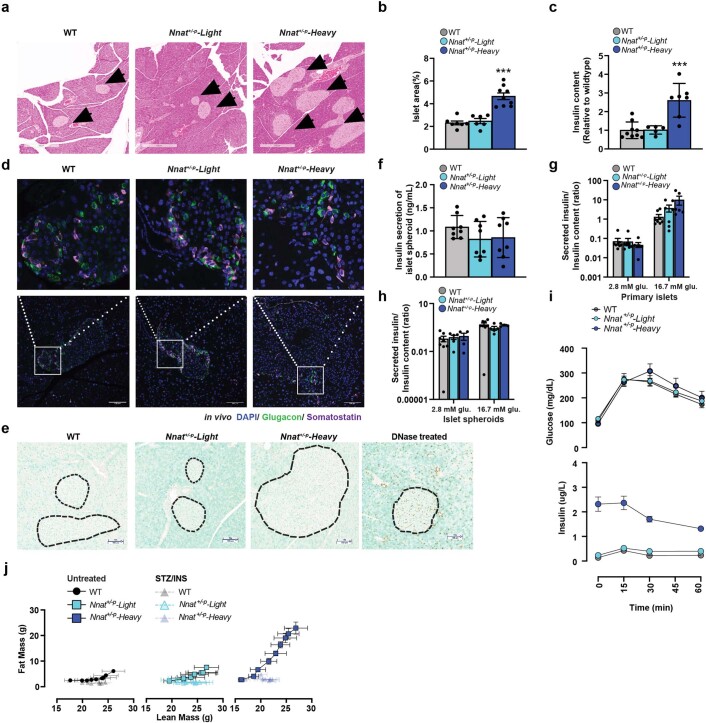
*Nnat*^*+/-*^*p*-Heavy morphs exhibit normal glucose tolerance and islet functionality. **a**, H&E staining was performed to locate islets (light pink areas, arrows) in *Nnat*^+^^*/-p*^ pancreata. Scale bar, 500 µm. The experiment was repeated independently 3 times with similar results. **b**, Islet area was quantified as percent of the entire pancreas area. Each group had 3 thin sections from at least 6 animals. All data were plotted as mean ± SEM, *** (adjusted *p* < 0.0001) by one-sided Tukey’s multiple comparisons test. **c**, Total insulin content was extracted from whole pancreata, normalized to total insulin in WT littermates. Each group had at least 5 animals. All data were plotted as mean ± SEM, *** (adjusted *p* = 0.0008) by one-sided Tukey’s multiple comparisons test. **d**, Glucagon and somatostatin staining was performed on *Nnat*^+^^*/-p*^ pancreata. Scale bar, 100 µm. The experiment was repeated independently 3 times with similar results. **e**, Cell death (apoptosis) event was examined via TUNEL assay in the pancreatic section from *Nnat*^+^^*/-p*^ and WT littermates at 16 weeks old. DNase treated sample as positive control from the same animals. The black circle heights the islet area. Each group had at least 3 animals. Scale bar, 100 μm **f**, Basal insulin secretion was measured from size-matched β-cell spheroids. Each group had 8 spheroids. All data were plotted as mean ± SEM. **g, h** Glucose-stimulated insulin secretion assays were performed on primary islets (**g**) and spheroids (**h**) from 16 weeks old *Nnat*^+^^*/-p*^ and WT littermates. 2.8 mM glu.: 2.8 mM glucose and 16.7 mM glu.: 16.7 mM glucose. At least 6 primary islets and 4 spheroids were in each group. All data were plotted as mean ± SEM. **i**, Oral glucose tolerance test (OGTT) was performed in 16 weeks old *Nnat*^+^^*/-p*^ and WT mice (n = 4-5) fasted for 6 hours and showed relatively normal glucose tolerance in *Nnat*^+^^*/-p*^-*Heavy* morphs. All data were plotted as mean ± SEM. **j**, Growth trajectories for untreated and STZ-treated *Nnat*^+^^*/-p*^ animals and WT littermates between 4 and 12 weeks of age. All data were plotted as mean ± SEM. Source data

We then analyzed morphology, function and turnover of the pancreatic islet β-cell compartment, the primary source of insulin in the body. *Nnat*^+/-p^-*Heavy* mice showed marked increases in β-cell mass (Fig. 2b and Extended Data Fig. 3a,b) relative to both WT and *Nnat*^+/-p^*-Light* groups. *Nnat*^+/-p^-*Heavy* animals also showed reduced insulin immunoreactivity (Fig. 2b), consistent with previous work showing that β-cell-specific loss of *Nnat* (in otherwise normal developmental contexts) impairs insulin secretion and storage^36^. Overall, *Nnat*^+/-p^-*Heavy* animals showed total pancreatic insulin content ~2.5-fold higher than WT and *Nnat*^+/-p^*-Light* groups (Extended Data Fig. 3c). Thus, *Nnat* deletion leads to distinct programming of β-cell mass in *Nnat*^+/-p^*-Light* and *Nnat*^+/-p^-*Heavy* mice.

*Nnat*^+/-p^-*Heavy* animals showed increased numbers of Ki-67-positive β-cells in vivo relative to *Nnat*^+/-p^-*Light* animals (Fig. 2c). No differences were observed in islet organization and rates of cell death (TUNEL) (Extended Data Fig. 3d,e). We therefore measured proliferation in islets ex vivo to validate these findings and assess the stability and cell autonomy of the hyperproliferative program. Increased β-cell proliferation was readily measurable in *Nnat*^+/-p^*-Heavy* islets after 3 days of ex vivo equilibration culture and after 2 days of 5-ethynyl-2'-deoxyuridine (EdU) incubation (Fig. 2d). These findings indicated islet-autonomous hyperproliferative programming in *Nnat*^+/-p^-*Heavy* animals. No measurable differences were observed in insulin release in both primary islets and reconstituted islet spheroids, under steady-state or glucose-stimulated conditions (Extended Data Fig. 3f–h). In line with these data, glucose tolerance was largely normal in *Nnat*^+/-p^-*Heavy* animals despite the marked hyperinsulinemia (Extended Data Fig. 3i). These data suggested that the overgrowth polyphenism is driven by an alternate β-cell hyperplasia program.

### *Nnat*^*+/-p*^ triggered overgrowth is insulin-dependent

To test whether probabilistic *Nnat*^+/-p^ overgrowth is driven by β-cell hyperplasia and resulting hyperinsulinemia, we artificially ‘clamped’ in vivo insulin levels at equal levels across groups by injecting animals with a single high-dose injection of streptozocin (STZ) to deplete the endogenous β-cell pool^44^ and implanting slow-release subcutaneous insulin pellets to restore insulin sufficiency equally across animals (Fig. 2e). Treatment was initiated at ~5 weeks of age in longitudinally tracked cohorts. Animals initiating their *Nnat*^+/-p^-*Heavy* trajectory (defined as a 3 g fat mass gain within 5 days) were randomly sorted into treatment or control groups. Notably and where parallel control cohorts of *Nnat*^+/-p^-*Heavy* morphs gained ~15 g of fat and ~3 g of lean mass beyond that of their WT and *Nnat*^+/-p^-*Light* siblings (Fig. 2f,g top; arrow from WT/crosshair), combination therapy completely abrogated this alternate phenotype, yielding lean and fat mass accumulations comparable to WT animals (Fig. 2f (bottom) and Extended Data Fig. 3j). All treated animals completed normal growth trajectories, reaching healthy mature body mass levels of ~30 g (Fig. 2g). Thus, *Nnat*^+/-p^-*Heavy* overgrowth is insulin-dependent.

### The alternate β-cell hyperplasia program is HDAC-dependent

**Fig. 3:**
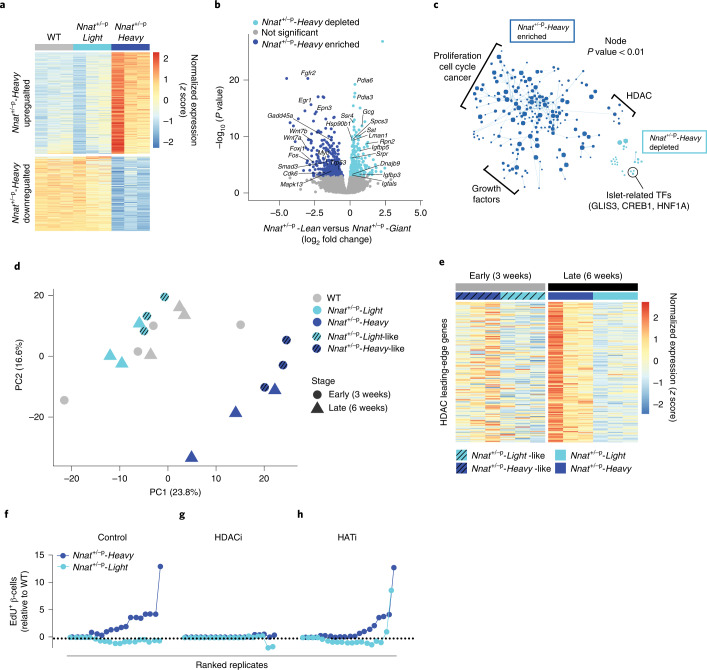
HDAC mediates *Nnat*^+/-p^-driven β-cell hyperplasia. **a**, Heat map showing the expression of 552 differentially expressed genes (DEGs), defined as *Nnat*^+/-p^-*Heavy* and *Nnat*^+/-p^-*Light* islets transcriptome, in the indicated animals. **b**, Volcano plot showing DEGs and highlighting the enriched or depleted biologically relevant genes in *Nnat*^+/-p^-Heavy morphs. *P* values as assessed by negative binomial generalized linear model. **c**, The cytoscape plot of the GSEA showed the enriched or depleted gene sets in *Nnat*^+/-p^-*Heavy* morphs. TFs, transcription factors. **d**, PCA demonstrated transcriptional similarity of *Nnat*^+/-p^-*Heavy*-like at early stages (3 weeks) and *Nnat*^+/-p^-*Heavy* morphs at late stages (6 weeks) apart from *Nnat*^+/-p^-*Light*-like at early stages (3 weeks) and *Nnat*^*+/-p*^-*Light* morphs at late stages (6 weeks). **e**, Heat map shows HDAC gene set leading-edge gene expression in *Nnat*^+/-p^-*Heavy*-like and *Nnat*^+/-p^-*Light*-like morphs (early stage) and *Nnat*^+/-p^-*Heavy* and *Nnat*^+/-p^-*Light* morphs (late stage). **f**–**h**, Proliferating β-cells were counted by EdU-incorporation from *Nnat*^+/-p^-*Heavy* and *Nnat*^+/-p^-*Light* morphs and are normalized to WT littermates in untreated (control, **f**), HDACi-treated (**g**) and HATi-treated (**h**) conditions. At least 19 islets were quantified and plotted per condition. Source data

**Extended Data Fig. 4:**
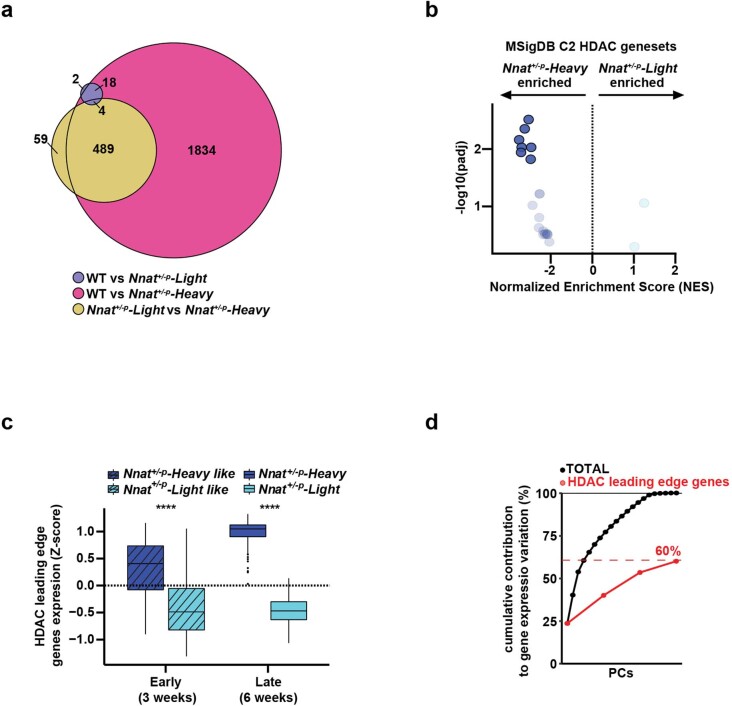
Dysregulated HDAC-related transcriptome precedes the *Nnat*^*+/-p*^-overgrowth. **a**, Venn diagram of differential gene expression analyses of islets transcriptome of 6 weeks old mice comparing between *Nnat*^*+/-p*^-Heavy, *Nnat*^*+/-p*^-Light morphs and WT littermates. **b**, GSEA results of HDAC-responsive gene sets between the *Nnat*^*+/-p*^-Light and *Nnat*^*+/-p*^-Heavy morphs, showing a specific enrichment in the latter. Solid and transparent colored dots, highlight either statistically significant or not significant enrichments, respectively (adjusted *p*-value cutoff < 0.05). **c**, Gene expression (Z-score) comparison was performed for HDAC gene set leading-edge genes between *Nnat*^*+/-p*^-Heavy-like and *Nnat*^*+/-p*^-Light-like morphs (early stage) and *Nnat*^*+/-p*^-Heavy and *Nnat*^*+/-p*^-Light morphs (late stage). **** (*p* ≤ 0.001) as assessed by two-tails *t*-tests. In all box-plots, the lower and upper hinges = 25th and 75th percentiles. The upper/lower whiskers = largest/smallest observation less/greater than upper/lower hinge + 1.5 * IQR. Central median = 50% quantile. **d**, Estimate of the contribution of the HDAC-responsive genes, to the overall transcriptional variability between WT, *Nnat*^*+/-p*^-Heavy and *Nnat*^*+/-p*^-Light mice. Following PCA, the dotplot shows either the cumulative contribution of all principal components (PCs) to gene expression variation (black dots/line), or the contribution of the top four PCs (red dots/line), mostly associated with the HDAC-leading-edge genes from (**b**). The cumulative contribution of these four PCs is describing 58.7% of total gene expression variation. Source data

To gain insight into the developmental switch driving β-cell hyperplasia in *Nnat*^+/-p^-*Heavy* morphs, we performed RNA-sequencing (RNA-seq) on *Nnat*^+/-p^ islets before and after the onset of detectable overgrowth bifurcation (3 and 6 weeks). Transcriptomes from WT and *Nnat*^+/-p^-*Light* islets showed minimal differences (Extended Data Fig. 4a), consistent with the phenotypic similarities between the two genotypes. These data demonstrate that whole-body *Nnat* deletion, by itself, is not sufficient to drive β-cell dysregulation and imply that *Nnat*’s primary function is to buffer against phenotypic variation. In contrast, *Nnat*^+/-p^-*Heavy* samples showed major transcriptional rewiring (Fig. 3a,b and Extended Data Fig. 4a). Consistent with the observed hyperplasia, gene set enrichment analysis (GSEA) revealed upregulation of pathways and leading-edge signatures associated with cell cycle (*Cdk6*, *Ccnl2*, *Myc* and *Tp53*), proliferation (*Wnt7a/b*, *Mapk13*, *Foxj1*, *Fos* and *Smad3*) and growth factor signaling (*Egr1, Fgfr2* and *Epn3*) (Fig. 3b,c). Downregulated factors included islet endocrine lineage hormone genes (*Gcg* and *Sst*), ER-processing (*Pdia3/4*, *Lman1/2*, *Rpn1/2*, *Hsp90b1*, *Dnajb9* and *Ssr4*) and protein export pathways (*Spcs3*, *Srps* and *Sec61b/g)* (Fig. 3b,c). Unexpectedly, *Nnat*^+/-p^-*Heavy* islets showed upregulation of a functionally disparate set of HDAC-responsive genes (Fig. 3c and Extended Data Fig. 4b). This result was particularly noteworthy because histone acetylation dynamics have been implicated in regulating insect polyphenisms^45,46^ and cell proliferation in cancer^47^.

To test whether HDAC regulation was causally linked to the overgrowth polyphenism, we first examined whether the HDAC transcriptional signatures were already present before the phenotypic bifurcation. Notably, the 3-week RNA-seq data revealed that *Nnat*^+/-p^ islet transcriptomes definitively separate into *Nnat*^+/-p^-*Light-*like or *Nnat*^+/-p^-*Heavy-*like clusters, before phenotypic distinctions are detectable (Fig. 3d). Fully 60% of the variation in the RNA-seq dataset correlated with the same HDAC-responsive gene sets (Fig. 3e and Extended Data Fig. 4c,d), indicating that the HDAC-associated genes are fundamentally responsible for the phenotypic variation in that moment of early life. The data also suggested that dysregulation of HDAC-responsive genes might cause the β-cell hyperproliferation phenotype in *Nnat*^+/-p^-*Heavy* animals. In either case, HDAC-associated transcriptional rewiring precedes the phenotypic bifurcation toward overgrowth.

Second, we cultured islets from the three genotype-phenotype combinations with histone acetylase inhibitors (HATi) or histone deacetylase inhibitors (HDACi) and tracked β-cell proliferation *in* vitro. Consistent with the data above, *Nnat*^+/-p^-*Heavy* islets showed islet-autonomous β-cell hyperplasia at baseline (Fig. 3f). Notably, HDACi treatment had no observable effect on WT and *Nnat*^+/-p^-*Light* islets, but the treatment was sufficient to abrogate *Nnat*^+/-p^-*Heavy* β-cell hyperplasia and return proliferation back to WT levels (Fig. 3g). These data demonstrate that HDAC-sensitive gene regulation is required for control of β-cell programming and that *Nnat*’s buffering effect on phenotypic variation is mediated through HDAC-responsive genes. HATi treatment, on the other hand, had no effect on *Nnat*^+/-p^-*Heavy* β-cells (Fig. 3h), indicating a necessary directionality to the regulatory process. Parallel treatment of *Nnat*^+/-p^-*Light* β-cells showed no substantial regulation by either inhibitor (Fig. 3f–h), further highlighting the specificity of the HDAC-dependence. Thus, *Nnat* buffers against probabilistic phenotypic variation by preventing the activation of an HDAC-dependent β-cell hyperplasia program.

### Identification of phenotypic variation patterns in humans

The foregoing data identify a *Nnat*-buffered axis that regulates probabilistic phenotypic variation. They demonstrate islet-autonomous underpinnings, reproducible epigenome dysregulation and reversibility at in vitro and organismal scales, all of which are unprecedented findings for a mammalian polyphenism. Critically, they demonstrate epistatic independence and additivity with *Trim28*-buffered phenotypic variation and thus identify two independent pathways for buffering alternate but bi-stable developmental trajectories and phenotypic heterogeneity.

In our previous work^43^, we found bimodal body mass distributions in large epidemiological cohorts, which raised the possibility that bi-stable UPV might also exist in human populations. That *Nnat* also buffers against bi-stable UPV again raised the question about regulatory control and polyphenism potential in humans. We therefore analyzed monozygotic (MZ) and dizygotic (DZ) twin data^8,48–52^ for potential signatures of human UPV. While twin analyses do not rule out genetic and environmental trait variation^5,53,54^, they substantially reduce these contributions. We performed a high-dimensional analysis of 35 anthropometric traits measured across 153 MZ co-twin pairs from the TwinsUK’s Multiple Tissue Human Expression Resource (MuTHER) cohort^55–57^. The high-dimensional approach served two purposes. First, the analysis makes no assumptions about how UPV should manifest, but instead hypothesizes that if regulated UPV systems exist in humans, their consequences should be reproducible. Second, searching for patterns of variation (twin discordance), as opposed to single-trait discordances, reduces the impact of measurement errors in any given trait.

**Fig. 4:**
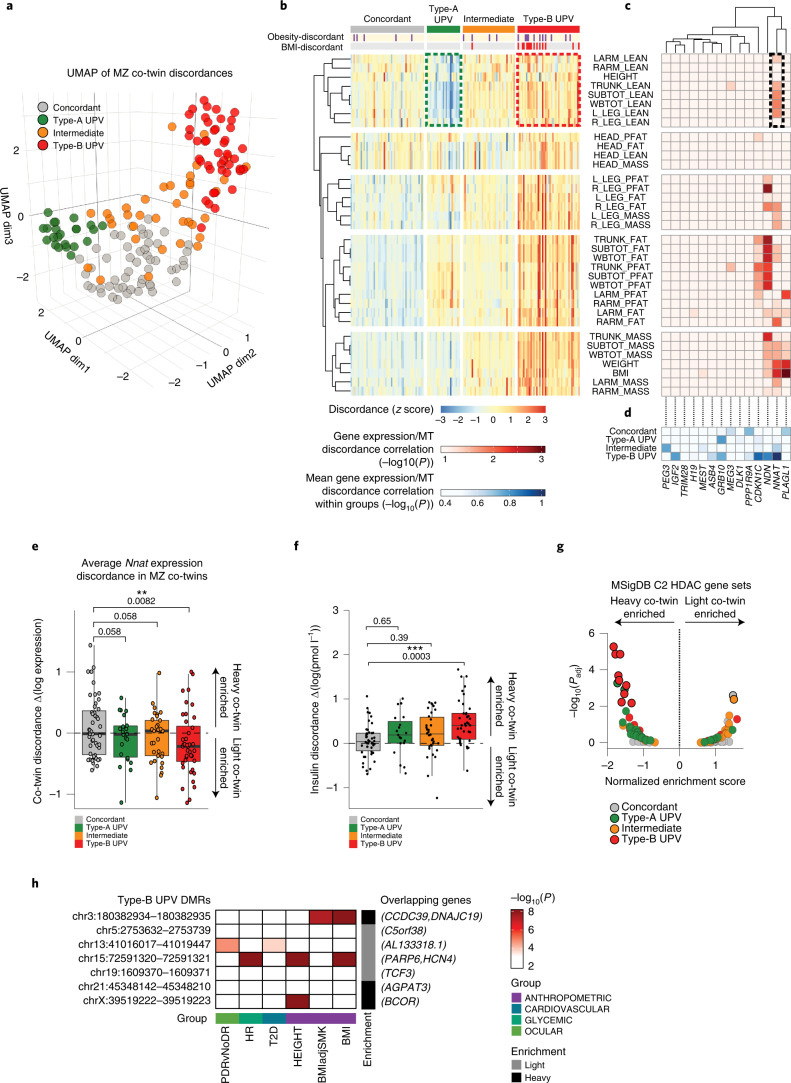
Characterization of human UPV. **a**, UMAP projection of MZ co-twin couples, according to 35 morphometric discordances. **b**, Heat map of hierarchical clustering of morphometric discordances among MZ co-twin couples. Obesity-discordant co-twins indicate that only one co-twin is affected by obesity (BMI > 30). BMI discordant co-twins, BMI difference >5 BMI points. Dashed colored boxes highlight distinct lean mass discordances between Type-A and Type-B UPV. **c**, Heat map showing the hierarchical clustering of *Trim28*/*IGN1* genes based on the correlation of their expression discordance and indicated phenotypic discordances. A dashed black box highlights *NNAT* expression discordance correlation with phenotypic discordances of those traits that distinguish Type-A and Type-B UPV. **d**, Heat map showing the hierarchical clustering of *Trim28*/*IGN1* genes based on the average correlation of their expression discordance and all phenotypic discordances, stratified by four co-twin pairs’ clusters. **e**, Box-plots representing discordance of *NNAT* expression, among MZ co-twins, belonging to the indicated clusters. **P = 0.0082, as assessed by one-tailed *t*-tests. **f**, Box-plots representing serum insulin discordance, among MZ co-twins, belonging to the indicated groups. ***P = 0.0003 as assessed by one-sided Tukey’s multiple comparisons test, following one-way analysis of variance (ANOVA). In all box-plots, lower and upper hinges indicate 25th and 75th percentiles. The upper/lower whiskers indicate largest/smallest observation less/greater than upper/lower hinge + 1.5 ×IQR. Central median indicates 50% quantile. **g**, GSEA results of HDAC-responsive gene sets between the ‘light’ and ‘heavy’ co-twins, belonging to the indicated MZ co-twins groups. Solid and transparent colored dots, highlight either statistically significant or not significant enrichments, respectively (adjusted *P* value cutoff <0.01). **h**, Heat map showing association of single-nucleotide polymorphisms (SNPs) and indicated phenotypic traits, within the DMRs identified between ‘light’ and ‘heavy’ Type-B UPV co-twins. White boxes indicate no significant associations (*P* > 1 × 10^−^^3^), dark-red boxes indicate genome-wide significant associations (*P* < 1 × 10^−8^). Nearest are reported. Gray and black boxes indicate the enrichment of DMR in either the ‘light’ or ‘heavy’ co-twin. BMIadjSMK, BMI adjusted by smoking; T2D, type 2 diabetes; HR, heart rate; PDR, proliferative diabetic retinopathy; PDRvNoDR, PDR versus no PDR. Source data

**Extended Data Fig. 5:**
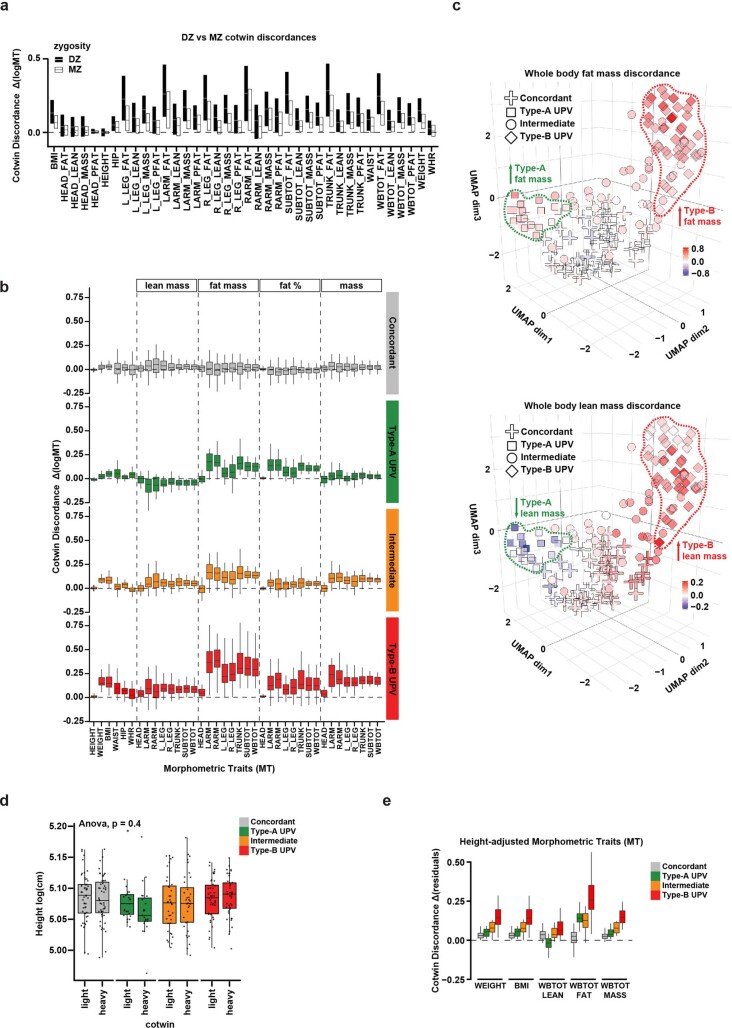
Characterization of the concordant/discordant MZ co-twin groups. **a**, Boxplot showing the inter-quartile ranges (IQR) of co-twin discordance indices for all the available features in either MZ or dizygotic (DZ) co-twins. **b**, Boxplot showing the MZ co-twin discordance indices for the indicated morphometric features, in the concordant/discordant groups identified. A horizontal dashed line highlights zero values of the discordance index (that is, concordance). Vertical dashed lines separate fat/lean/total mass and percentage of fat from the indicated body parts. **c**, UMAP projection of MZ co-twin couples from TwinsUK (n = 153), according to 35 morphometric discordances. Each observation represents a twin pair, colored by the whole-body fat (above) or lean (below) mass discordance (calculated as the difference between log-transformed measurements). The different shapes of the co-twin pairs represent the identified groups, as indicated. Dotted red/green lines highlight the Type-A and Type-B UPV and show their differences with respect to lean mass discordance. **d**, Box-plots representing the average height, between the ‘light’ and ‘heavy’ MZ co-twins, belonging to the indicated groups. Solid horizontal lines represent medians. The *p*-value is calculated by ANOVA. **e**, Boxplot showing the MZ co-twin discordance indexes for the indicated morphometric features, after height-normalization, in the concordant/discordant groups identified. A horizontal dashed line highlights the zero value of the discordance index (that is, concordance). Box plots in (**b**,**d**,**e**) show the lower and upper hinges = 25th and 75th percentiles. The upper/lower whiskers = largest/smallest observation less/greater than upper/lower hinge + 1.5 * IQR. Central median = 50% quantile. Source data

We calculated co-twin trait discordance for each trait, which included weight, height and fat and lean masses of the head, trunk and limbs. Discordance between each trait was calculated for each co-twin pair, setting the co-twin with the lower body mass index (BMI) as the reference. Discordances for all traits were uniformly lower in MZ co-twins than DZ co-twins, as expected (Extended Data Fig. 5a). We then focused on MZ twin pairs and performed a Uniform Manifold and Projection (UMAP) dimensional reduction^58^ on the 35 trait discordance × 153 co-twin pair matrix (Fig. 4a). Notably, the analysis revealed four clusters or ‘patterns’ of phenotypic variation in the cohort: two were distinctly discordant clusters (Type-A (green) and Type-B (red); Fig. 4a,b) and one was a central concordant cluster (gray). An ‘Intermediate’ UPV cluster (orange) was also identified tending toward Type-B UPV. Thus, we identified four candidate patterns of human UPV.

**Extended Data Fig. 6:**
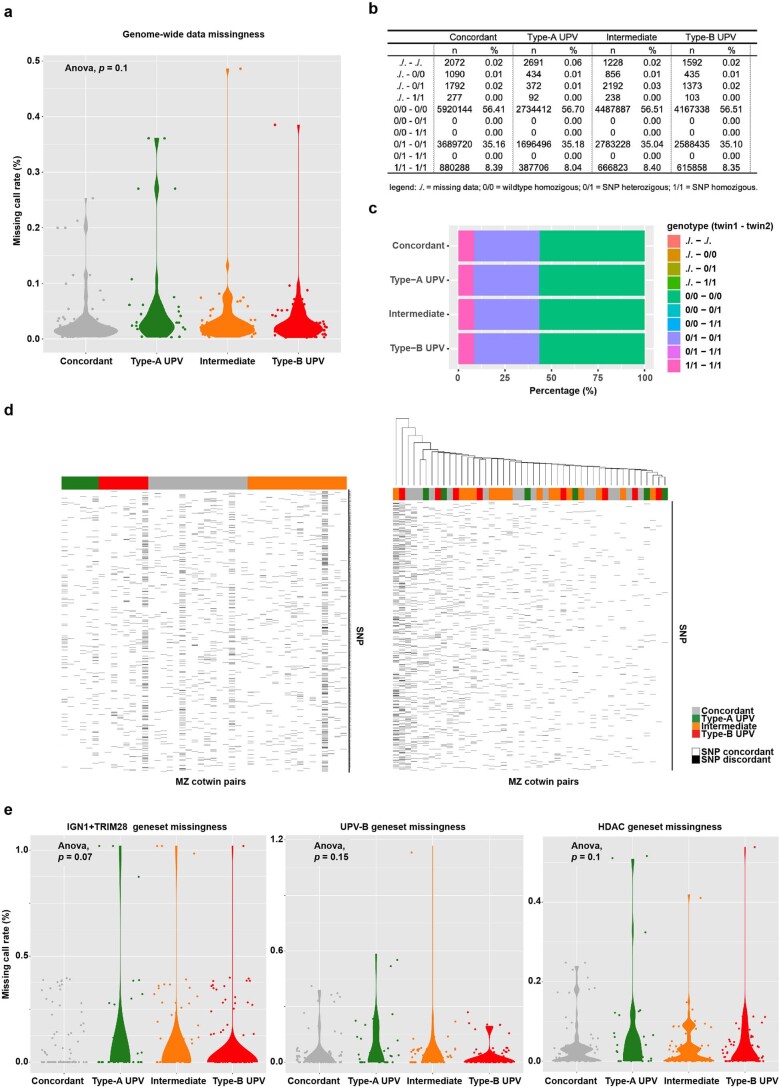
UPV groups are not determined by genetic differences between MZ cotwins. **a**, Violin-plots showing the distribution of missing data (as percentage of the total) among individuals’ genotypes, stratified by UPV groups. This data show that the degree of genome wide missingness is not correlated to UPV groups. **b**, Table summarizing the amount of genetically identical *loci* among MZ cotwins and genetic differences, only due to missing data. **c**, Barplot summarizing the data in table b. MZ cotwins were identical on > 99.9% of the analyzed *loci* and differences due to missing data account on average for ~ 0.06% of the total data, among all UPV groups. **d**, Heatmaps showing the distribution of SNPs that resulted different between MZ cotwins, only due to missing data. On the left, the cotwin pairs are ordered by the UPV sub-types. On the right, they are ordered according to hierarchical clustering. These data show that neither the degree of missingness, nor the specific genomic positions of missing data showed any correlation to UPV sub-types. **e**, Same as in a, on the indicated genesets. These data show no specific enrichment of missingness in any UPV group, nor any genesets, arguing against evidence for genotypic differences underlying the detected transcriptional signatures. All *p*-values as assessed by ANOVA.

The concordant cluster was characterized by minimal co-twin trait discordance across all traits, which is what we would normally expect from stereotypical ‘identical twins’ (Fig. 4b and Extended Data Fig. 5b). Type-A UPV was characterized by increased fat masses and a modest reduction of lean masses in heavier co-twins (dashed green box; Fig. 4b and Extended Data Fig. 5b,c). By contrast, Type-B UPV was characterized by increases in both fat and lean masses across body parts in the heavier co-twin (Fig. 4b, dashed red box and Extended Data Fig. 5b,c). These two patterns were distinct and identified an inherently reproducible substructure in ‘non-genetic’ human variation. The findings provided a refined view of twin variation relative to arbitrary BMI and obesity cutoffs^59,60^ (Fig. 4b; for example ‘BMI’ and ‘obesity’ discordance top annotations). No differences were observed in mean height or height discordance across clusters (Extended Data Fig. 5b,d) and repeat analysis using height-adjusted trait discordances captured the same fundamental UPV patterns (Extended Data Fig. 5e). Notably, we also analyzed genotyping data across the individuals of our four identified UPV groups of MuTHER UK twins^57^. We found no evidence of consistent genotypic differences between MZ co-twins that extended beyond expected data missingness and that could conceivably underlie the observed patterns (Extended Data Fig. 6a–e). Thus, we identified two ‘non-genetic’ patterns of human phenotypic variation, Type-A UPV, characterized by reciprocal fat and lean mass differences (a relative adiposity) and Type-B UPV, characterized by coordinated fat and lean mass dysregulation (a relative overgrowth).

### Type-B human UPV parallels *Nnat*^*+/-p*^*-Heavy* overgrowth

We next explored adipose tissue transcriptomic data from the same co-twin pairs and asked whether *NNAT* expression was associated with any of the human UPV clusters. Correlative analysis revealed substantial correlations between expression of several *IGN1* genes and trait discordances, including *NNAT*, *NDN*, *CDKN1C* and *PLAGL1* (Fig. 4c). Of note, *NNAT* was the only gene whose expression discordance consistently correlated with both fat and lean mass discordances, features that were also specific to Type-B UPV (Fig. 4c; dashed box). Indeed, when data were stratified by UPV sub-type, *NNAT* expression associated strongly with trait discordance specifically in Type-B UPV (Fig. 4d) with expression decreased in heavier Type-B UPV co-twins (Fig. 4e). These results suggest that *NNAT* discordance (and downregulation) is a hallmark signature of Type-B human UPV. *TRIM28* expression showed no correlation with trait discordances at either the cohort or UPV sub-type levels (Fig. 4c,d). Thus, *NNAT* gene expression associates with Type-B human UPV.

**Extended Data Fig. 7:**
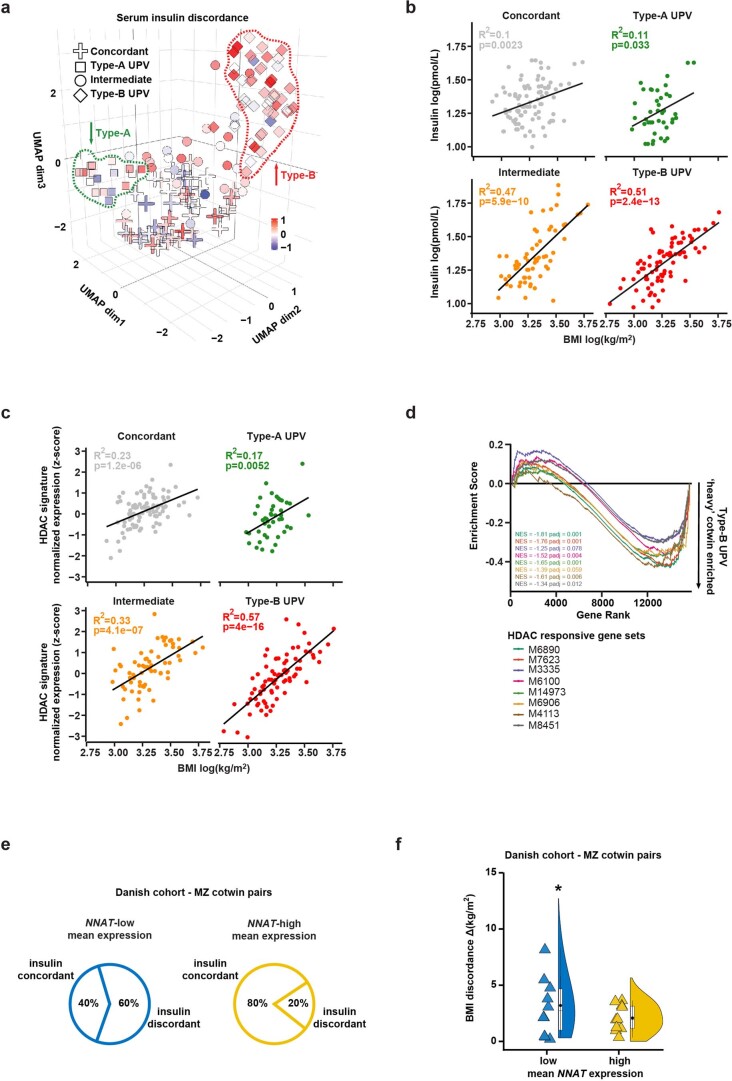
Type-A and Type-B UPV show specific metabolic and molecular profiles. **a**, UMAP projection of MZ cotwin couples form the TwinsUK (n = 153), according to 35 morphometric discordances. Each dot represents a cotwin pair, colored by their serum insulin level discordance (calculated as the difference between log-transformed measurements). The different shapes of the cotwin pairs represented the identified groups. Dotted red/green lines highlight the Type-A and Type-B UPV and show the increase insulin levels (that is, relative hyperinsulinemia) in Type-B ‘heavy’ cotwins. **b,c**, Scatterplots showing the correlation between BMI and insulin level (**b**) or the HDAC-signature expression (**c**), in the indicated cotwin groups. Both showing stronger correlation in the Type-B UPV cotwins (BMI/insulin: R^2^ = 0.51, *p*-value=2.4^-13^, BMI/insulin: R^2^ = 0.57, *p*-value=4^-16^). R^2^ and *p*-values as calculated by fitted linear regression models. **d**, Gene set enrichment analysis (GSEA) results of HDAC-related gene sets between the ‘light’ and ‘heavy’ cotwins, belonging to the Type-B UPV group. Gene sets specifically enriched in ‘heavy’ cotwins of the Type-B UPV cluster are shown. NES = normalized enrichment score; padj = adjusted *p*-value. **e**, Pie-charts, showing the distribution of insulin-concordant/discordant MZ cotwin pairs, from the Danish twin cohort, in either low- (blue chart), or high-*NNAT* expressing couples (yellow chart). The definition of insulin-concordant/discordant couples was obtained by Gaussian finite mixture modeling (see Methods section). **f**, Box- and violin-plots of BMI discordance distributions, represented as inter co-twin differences in the Danish twin cohort. Solid horizontal lines and black points represent means and medians in the box-plots, respectively. * *p*-value = 0.03, as assessed by the Bartlett’s test of homogeneity of variances. Blue and yellow triangles represent co-twin pairs expressing averaged low and high NNAT levels, respectively. In box-plots, the lower and upper hinges = 25th and 75th percentiles. The upper/lower whiskers = largest/smallest observation less/greater than upper/lower hinge + 1.5 * IQR. Central median = 50% quantile. Source data

Given this clear and specific association between *NNAT* expression and Type-B UPV, we tested whether other key features of the murine overgrowth polyphenism were also altered in human Type-B UPV. For insulinemia, we found that plasma insulin discordances were highest in Type-B co-twin pairs (Fig. 4f and Extended Data Fig. 7a). Indeed, the Type-B UPV group exhibited the clearest correlation between insulinemia and BMI levels (*R*^2^ = 0.51; *P* = 2.4 × 10^−13^), an association that extended across the BMI spectrum (Extended Data Fig. 7b) and was well above the concordant and Type-A UPV groups (*R*^2^ = 0.1 and *R*^2^ = 011, respectively). These data indicate that Type-B UPV represents a unique metabolic state where insulinemia and BMI are tightly coupled. Similarly, we found a substantially more robust HDAC-target gene regulation associated with BMI specifically in Type-B UPV (*R*^2^ = 0.57; *P* = 4 × 10^−16^; Extended Data Fig. 7c). Consistent with the directionality of the *Nnat*^+/-p^ mouse system, GSEA analysis showed that HDAC-target genes were upregulated in heavier Type-B co-twins (Fig. 4g and Extended Data Fig. 7d). Thus, hyperinsulinemia, HDAC-target gene upregulation and lean and fat mass excesses are species-conserved features of *NNAT*-associated UPV. From the clinical perspective, these data suggest a model where *NNAT* buffers against emergence of a distinct phenotypic state (polyphenism) where BMI is exceptionally coupled to insulinemia, lean and fat mass excess.

To validate these findings, we examined adipose *NNAT* gene expression levels and available anthropomorphic traits in a small, independent Danish twin cohort. The cohort consisted of 20 MZ same-sex co-twin pairs^61^. As the cohort size and available traits precluded the same clustering analysis (as in Fig. 4), we stratified the cohort into upper and lower quantiles of *NNAT* expression. Notably, we validated the key findings from the MuTHER cohort: we observed increased insulinemia discordance (Extended Data Fig. 7e) and increases in BMI discordance specifically in MZ co-twin pairs with low *NNAT* expression (Extended Data Fig. 7f). Thus, reduced *NNAT* expression is associated with increased phenotypic variation in BMI and insulinemia in an independent twin cohort.

### Human UPV sub-types are epigenetically distinct

**Extended Data Fig. 8:**
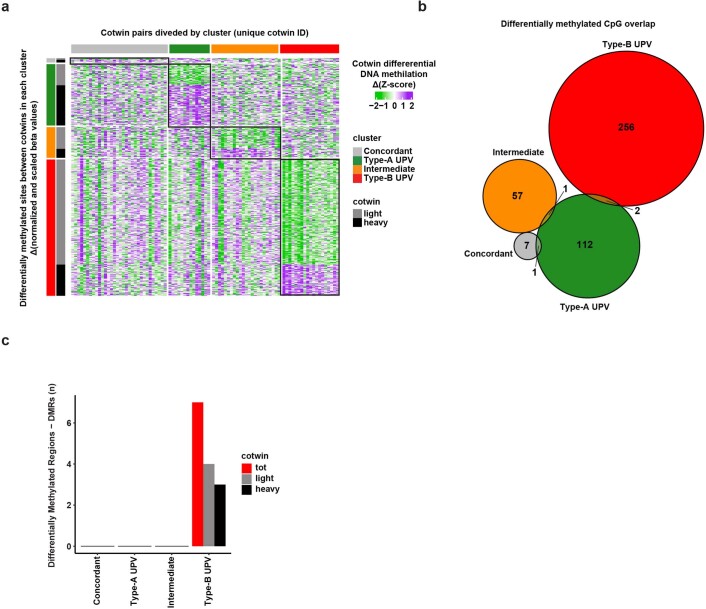
Type-A and Type-B UPV are associated to distinct DNA methylation patterns. **a**, heat map showing the differentially DNA methylated sites among ‘heavy’ and ‘light’ cotwins belonging to the indicated groups identified in the TwinsUK cohort. SeSAMe cutoffs: adjusted *p*-value < 0.05; effect size > 0.05. Dark-gray and black boxes highlight DNA methylation enrichment in either the ‘light’ or ‘heavy’ cotwin, respectively. **b**, Venn diagram showing the overlap between differentially DNA methylated sites from the indicated cotwin groups and highlighting the specificity of these epigenetic profiles. **c**, Barplots showing the amount of differentially methylated regions (DMRs) among ‘heavy’ and ‘light’ cotwins belonging to the indicated groups identified in the TwinsUK cohort. Only in Type-B UPV, DMRs were detected. Dark-gray and black bars highlight DNA methylation enrichment in either the ‘light’ or ‘heavy’ cotwin, respectively. Source data

Altered epigenetic control is believed to be a key mediator of developmental programming effects^62,63^. We tested whether any of our human UPV types showed evidence of unexpected epigenetic alteration. We analyzed Infinium HumanMethylation450 DNA methylation profiles from the same MuTHER cohort adipose samples and called differentially methylated sites between heavy versus light co-twins within each of the four UPV groups (Extended Data Fig. 8a). Concordant co-twins had the fewest differentially methylated sites (Extended Data Fig. 8a,b). Type-A and Type-B discordant co-twins exhibited hundreds of differential sites, with Type-B UPV showing the most differently methylated sites (Extended Data Fig. 8a,b). Whereas Type-A and Type-B UPVs are both characterized by relative increases in fat mass (they are both relative adiposities), their adipose tissue DNA methylation profiles were clearly distinct. On average, Type-A UPV was characterized by DNA hypermethylation in the heavier co-twin, as opposed to DNA hypomethylation in Type-B UPV (Extended Data Fig. 8a). Further, the differentially methylated sites of Type-A and Type-B discordant co-twins showed almost no overlap, indicating that the two types of human phenotypic variation are truly and fundamentally distinct in their nature (Extended Data Fig. 8b). Consistent with this idea, a search for differentially methylated regions (DMRs), as opposed to differentially methylated sites, identified only DMRs between co-twins of the Type-B UPV group (Extended Data Fig. 8c). Seven DMRs were identified that reproducibly distinguish the heavy and light Type-B co-twins. Four of the seven Type-B DMRs were hypomethylated in the heavy co-twin. Notably, these DMRs were enriched for proximity to significant genome-wide association study (GWAS) variants for BMI, height, body fat percentage, insulin sensitivity, insulinogenic index, diabetes and diabetes-associated cardiovascular disease (Fig. 4h). These findings directly link *NNAT* and Type-B UPV to epigenetic changes near causal, metabolic disease loci. Of the 15 genes neighboring the DMRs, a substantial fraction is involved in energy metabolism (*PARP6* and *AGPAT3*), transcriptional and epigenetic regulation (*TCF3* and *BCOR*) and associated either directly or indirectly to inherited syndromes causing lipodystrophy, hypotonia and intellectual and heart disorders (*C5orf58*, *AGPAT3*, *DNAJC19* and *HCN4*). Thus, human Type-B UPV is characterized by epigenetic regulation near human metabolic disease *loci*.

### Type-B UPV stratifies human into distinct metabolic subgroups

**Fig. 5:**
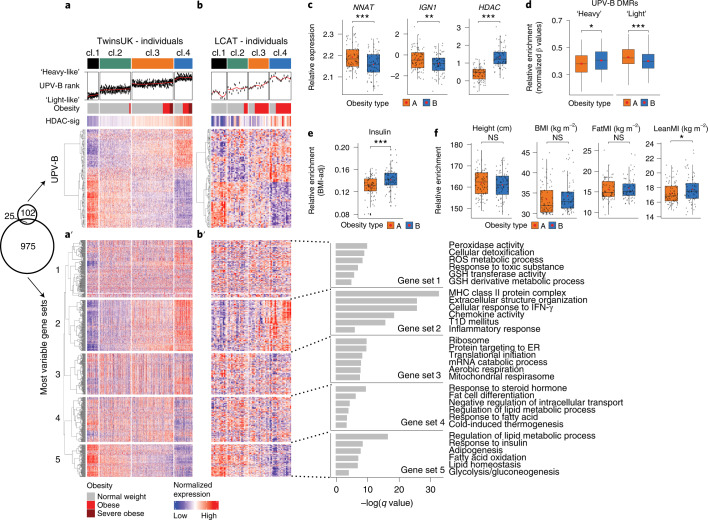
Type-B UPV signature separates adults and children into distinct phenotypic and metabolic sub-types. **a**, Heat map of *k*-means clustering of TwinsUK individuals. Four clusters were generated according to expression of the UPV-B signature. The UPV-B ranks annotation show the median rank of everyone according to their level of expression of UPV-B signature genes, discriminating Type-B ‘heavy-like’ and ‘light-like’ individuals. The obesity annotation is based on arbitrary cutoffs of BMI (obesity, >30 BMI; severe obesity, >35 BMI). The average expression of HDAC-signature (HDAC-sig) genes (leading-edge genes from Extended Data Fig. 6d) is reported. **a′**, Heat map (bottom), the expression profile of the most variable genes (top 1,000) across all samples is reported, after *k*-means clustering into five gene sets. Venn plot (left) shows the overlap between the most variable genes and the UPV-B. **b–b′**, Same as in **a**–**a′**, but on the LCAT cohort. The obesity annotation is based on standardized BMI arbitrary cutoffs (BMI standard score (SDS), obesity >1.88). On the right, representative results from Gene Ontology (GO) and pathway enrichment analysis for the five gene sets from the heat map of the TwinsUK individuals (**a′**). GO, KEGG and Molecular Signatures Database (MSigDB) databases were assessed. Related to the extended analysis in Extended Data Fig. 8g. **c**–**f**, Box-plots showing the distributions of indicated gene expression profiles (**c**), normalized DNA methylation on UPV-B DMRs (**d**), metabolic traits (**e**) and morphometric measurements (**f**), between Type-A and Type-B obesities (TwinsUK individuals affected by obesity and belonging to clusters 3 and 4 (cl.3 and 4) from the heat map in **a**). *P ≤ 0.05, **P ≤ 0.01, ***P ≤ 0.001, NS, not significant, as assessed by two-tailed Student’s *t*-tests. *NNAT* *P* = 0.00036; *IGN1* *P* value = 0.0067; *HDAC* *P* = 2.2 × 10^–16^; UPV-B DMRs ‘heavy’ *P* = 0.028; UPV-B DMRs ‘light’ *P* = 0.00026; insulin *P* = 0.001; height *P* = 0.4; BMI *P* = 0.21; FatMI *P* = 0.46; LeanMI *P* = 0.047. In all box-plots, the lower and upper hinges indicate 25th and 75th percentiles. The upper/lower whiskers indicate largest/smallest observation less/greater than upper/lower hinge + 1.5 × IQR. Central median indicates 50% quantile. GSH, glutathione; MHC, major histocompatibility; IFN, interferon; T1D, type 1 diabetes. Source data

**Extended Data Fig. 9:**
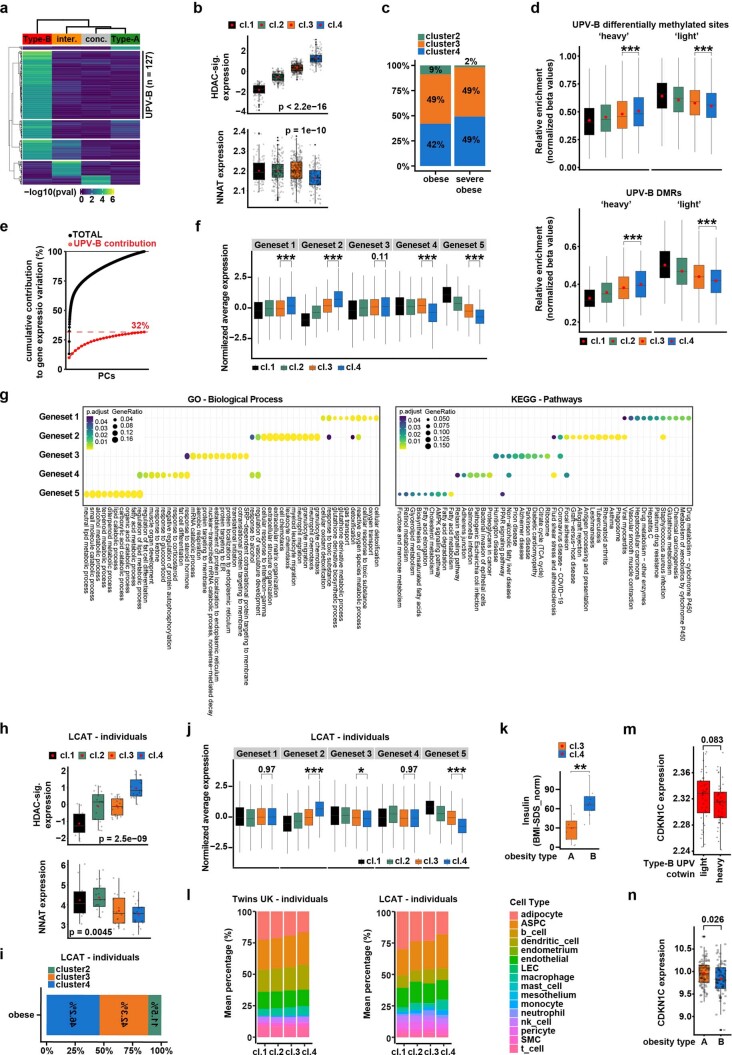
Characterization of adults’ and childrens’ clusters. **a**, Heat map of hierarchical clustering of DEGs between cotwins belonging to indicated groups. **b**, Boxplot showing the mean expression of HDAC-signature (above) or *NNAT* (below), among indicated clusters. *p*-values (2.2e-16; 1e-10) from ANOVA. **c**, Barplot showing obesity distribution among clusters. **d**, Boxplot showing normalized beta values for differentially methylated sites (above, p = 2.2e-16 and 2.2e-16) or regions (below, p = 0.0005 and 2.6e-8), among cotwins. ‘Heavy’- and ‘light’ enriched DNA methylated sites are reported. *** (*p* ≤ 0.001) from two-tails *t*-tests. **e**, UPV-B genes estimate of contribution to overall transcriptional variability in the TwinsUK cohort. Cumulative contribution of all principal components (black dots/line) and contribution of the most UPV-B-associated PCs (red dots/line) are reported. **f**, Boxplot showing mean expression of genes belonging to five genesets form Fig. 5b, among clusters. *** (*p* = 2.2e-16) from one-sided Tukey’s multiple comparisons test, following significant ANOVA. **g**, Complete gene ontology and pathway enrichment analysis for the 5 genesets from the heat map of TwinsUK individuals. **h**, Boxplot showing mean expression of HDAC-signature (above) or *NNAT* (below), among the clusters of the LCAT cohort. *p*-values from ANOVA. **i**, Barplot showing obesity distribution, among cluster in the LCAT cohort. **j**, Boxplot showing mean expression of genes belonging to the five genesets form Fig. 5b, among clusters in the LCAT cohort. * (*p* = 0.004), *** (*p* = 2.2e-16) from one-sided Tukey’s multiple comparisons test, following significant ANOVA. **k**, Box-plots showing distributions of serum insulin levels normalized on BMI-SDS, among children with obesity belonging to indicated clusters. ** (*p* = 0.0016) from two-tails *t*-tests. **l**, Barplots showing average cell-type compositions among transcriptional profiles of individuals belonging to indicated clusters. ASPC = adipose stem and progenitor cells, LEC = lymphatic endothelial cells, SMC = smooth muscle cells. **m-n**, Box-plots showing *CDKN1C* expression between ‘light’ and ‘heavy’ Type-B UPV cotwins (**m**) and Type-A/-B obesities (**n**). In all box-plots, lower and upper hinges = 25th and 75th percentiles. The upper/lower whiskers = largest/smallest observation less/greater than upper/lower hinge + 1.5 * IQR. Central median = 50% quantile. *p*-values from one-tail (**m**) or two-tails (**n**) *t*-tests. Source data

The analysis above showed that at least 30% of twin pairs in the MuTHER cohort exhibited Type-B UPV. If Type-B UPV is truly so common, then Type-B signatures should be readily detectable in the general population. We tested this idea in the greater MuTHER cohort, which includes hundreds of other individuals^56,57^ (Fig. 5a). First, we generated a Type-B UPV gene expression signature by identifying the 127 genes differentially regulated between heavy and light co-twins in the Type-B phenotypic variation cluster, but not differentially regulated between co-twins of the three other phenotypic variation clusters (Extended Data Fig. 9a and Methods). We then used hierarchical clustering to stratify the cohort according to expression of these 127, Type-B UPV genes. This analysis revealed four reproducible clusters of individuals in the general population (clusters 1–4 top, Fig. 5a). Individuals in clusters 2 and 3 showed little if any coordinated regulation of Type-B UPV genes (Fig. 5a). Clusters 1 and 4, however, showed strong coordinated regulation of Type-B UPV genes (Fig. 5a; heat map and rank analysis (UPV-B rank)). Cluster 4 individuals were specifically enriched in ‘heavy-like’ UPV-B transcriptome signatures, whereas cluster 1 individuals had ‘light-like’ gene expression profiles (Fig. 5a, UPV-B rank). Notably, clusters 1 and 4 also exhibited anti-correlated expression of *NNAT* (Extended Data Fig. 9b) and the non-overlapping HDAC-responsive genes. The latter indicated that the Type-B UVP gene signature (127 genes) captures the *NNAT*-buffered axis of phenotypic variation observed in the mouse and Type-B co-twins (Fig. 5a, HDAC-signature and Extended Data Fig. 9b). Thus, we find molecular evidence for Type-B human phenotypic variation (or plasticity) in the general population.

Consistent with their ‘light-like’ gene expression signature, cluster 1 individuals were all not affected by obesity (Fig. 5a, obesity annotation (top) and Extended Data Fig. 9c). In contrast, cluster 4 was enriched for individuals with obesity (Fig. 5a, obesity annotation (top) and Extended Data Fig. 9c). What is even more notable, is that cluster 4 still included many individuals without obesity (~50% of the cluster) despite strong Type-B UPV and *NNAT*/HDAC gene expression signatures (Fig. 5a and Extended Data Fig. 9c). These data are consistent with the fact that approximately 62% of Type-B ‘heavy*’* co-twins were also not affected by obesity (BMI < 30). Along similar lines, there was no correlation between Type-B gene expression and BMI within population clusters (Fig. 5a; data within clusters are ordered from low to high BMI). Examination of Type-B differentially methylated sites and DMRs showed a clear enrichment of heavy-like methylation patterns in cluster 4 individuals (Extended Data Fig. 9d). Thus, Type-B UPV captures a dimension of population-level variation that is distinct from obesity per se (Fig. 4b).

### Type-B UPV shows an inflammatory transcriptional program

Principal-component analysis (PCA) revealed that approximately one-third of all variation in the transcriptional dataset correlates tightly with the Type-B UPV gene expression signature (Extended Data Fig. 9e). This indicates that a major dimension of population-level phenotypic variation can be attributed to the Type-B ‘state’. To better understand the potential health implications of this finding, we analyzed the 1,000 most variable genes in the same transcriptional dataset. Of note, these 1,000 genes were distinct from the 127-gene Type-B UPV signature itself (Fig. 5a; Venn diagram). Consistent with the cumulative PCA analysis, two of the five major groups of the 1,000 most variable genes correlated strongly with the Type-B UPV gene signature (Fig. 5a′; gene sets 2 and 5). Cluster 4 (UPV-B heavy-like) individuals expressed high levels of inflammatory, immune and reactive oxygen species (ROS)-associated genes (Fig. 5a′, gene set 2, and Extended Data Fig. 9f,g). Conversely, the same individuals showed downregulation of genes involved in adipogenesis, lipid and glucose homeostasis (Fig. 5a′, gene set 5, and Extended Data Fig. 9f,g). Of note and as with the Type-B UVP gene signature itself, gene sets 2 and 5 were not correlated with obesity within clusters. These data again argue that the phenotypic outcomes of *NNAT* downregulation and Type-B UPV are not obesity per se. Rather, Type-B UPV consists of an altered metabolic state characterized by enhanced adipose tissue inflammatory signatures and reduced adipogenesis, nutrient uptake and metabolism pathway expression. Notably, cluster 4 included both individuals with and without obesity. Type-B UPV therefore also captures what seems to be a clear group of ‘normal weight obesity’ (individuals with normal BMI but with hyperinsulinemia and a highly consistent inflammatory gene expression pattern in adipose tissue).

### Type-B UPV is present in childhood

Guided by the fact that we identified Type-B UVP in controlled genetic contexts (isogenic *Nnat*^+/-p^ mice and MZ twins) and that the Type-B gene signature exhibited the most striking and distinct epigenetic characteristics (HDAC-signatures and DNA methylation signatures), we reasoned that Type-B phenotypic variation represents a state of altered epigenetic programming initiated in early life^64^. We therefore repeated our analysis using the Leipzig Childhood Adipose Tissue (LCAT) cohort^65^, a childhood cohort that had equivalent (and relevant) phenotypic and adipose tissue transcriptomic data (Fig. 5b). Notably, this analysis recapitulated all key findings above. The Type-B UPV gene expression signature stratified the childhood cohort into four corresponding clusters: cluster 4 children showed the strongest Type-B, heavy-like gene expression patterns (Fig. 5b) and cluster 1 showed the least. Cluster 4 children showed the strongest enrichment of HDAC-responsive genes (Fig. 5b (top) and Extended Data Fig. 9h) and reduced *NNAT* expression (Extended Data Fig. 9h). As with the adult cohort, all clusters included lean individuals; clusters 3 and 4 were enriched for individuals with obesity (Extended Data Fig. 9i); and while cluster 4 was the mostly enriched for obesity, it again included a substantial number of lean individuals. Also, in line with the adult analysis, cluster 4 children showed increased expression of inflammatory genes (Fig. 5b′, gene set 2, and Extended Data Fig. 9j) and downregulation of the lipid metabolism and adipocyte-specific genes (Fig. 5b′, gene set 5, and Extended Data Fig. 9j). Thus, Type-B UPV is readily observed in childhood and stratifies children into comparable metabolic ‘states’.

### Type-B UVP gene expression identifies two major obesity types

One of the most relevant implications of these data is that they suggest the existence of fundamentally distinct metabolic disease sub-types and in particular, two major sub-types of obesity. To test this idea, we performed a focused comparison of the individuals with obesity in clusters 3 and 4 (Type-B UPV gene signature stratified) (Fig. 5c–f). Relative to cluster 3 obesity, cluster 4 individuals with obesity showed enrichment of the Type-B UPV gene expression signature (Fig. 5a, heat map and rank analysis (UPV-B rank)); reduced *NNAT*/*IGN1* expression (Fig. 5c); increased HDAC-responsive gene expression (Fig. 5c); increased inflammatory and decreased adipogenesis gene expression (Fig. 5a′, gene set 2 and 5); and marked dysregulation of Type-B-specific DMRs (Fig. 5d). Phenotypically, cluster 4 individuals with obesity also exhibited consistently higher serum insulin levels (Fig. 5e); and while not different in height, BMI or relative fat mass (fat mass index; FatMI), cluster 4 individuals with obesity showed increased relative lean mass (lean mass index; LeanMI) (Fig. 5f). The distinctions between cluster 3 and cluster 4 obesities were validated in the LCAT childhood cohort for all available parameters (Fig. 5b,b′, gene sets and Extended Data Fig. 9k). Notably, this finding suggests that the pathophysiological consequences of Type-B UPV can already arise in early life. The data show that cluster 3 and cluster 4 obesities are distinct from phenotypic, transcriptional and epigenetic points of view. Given the parallels to co-twin-based Type-B UPV, we refer to these as Type-A obesity (cluster 3) and Type-B obesity (cluster 4).

Finally, to gain deeper insight into the transcriptional signatures themselves and their clinically relevance, we performed cell-type deconvolution using CibersortX^66^ and a recently published human adipose tissue single-cell atlas^67^. The analysis, which derives estimates of relative cell-type content in the MuTHER and LCAT cohort adipose biopsies, revealed high consistency across groups and cohorts (Extended Data Fig. 9l). Cluster 4, notably, showed a relative increase in pre-adipocyte-to-adipocyte ratio relative to cluster 3 and increased estimates for macrophage and monocyte content. These data are in line with the observed transcriptional signatures and indicated that Type-B (obesity and ‘normal weight obesity’) are characterized by increased adipose tissue inflammation and an adipocyte compartment skew toward adipose tissue progenitors.

Thus, we identify two major forms of obesity over and above the distinct classes of human phenotypic variation.

## Discussion

UPV is pervasive in complex traits and disease, even among highly inbred animals and MZ twins. Yet we have very limited understanding of the regulatory mechanisms underpinning UPV. In precision medicine and according to canonical models, UPV is typically attributed to uncharacterized early (developmental programing) or later-life environmental effects or dismissed as random biological ‘noise’. Here, we demonstrate the existence of a conserved, *NNAT*-regulated axis that buffers against phenotypic variation (as a trait) and control an overgrowth polyphenism. We show that the same axis stratifies human populations into unique metabolic classes and two common obesity sub-types that are distinct in their clinical presentation, transcriptional signatures and epigenetic control. The data provide support for probabilistic, multi-stable phenotypic canalization events being a major driver of metabolic outcomes.

One key translational question raised by these data is whether *NNAT*-associated phenotypic variation and bi-stable effects are influenced by genetic or environmental interactions. Since our original description^43^, several groups^41,42^ generated anecdotal evidence for the existence of genetic and environmental modifiers of *NNAT*-dependent buffering of UPV. We have directly tested and validated the idea. We observe bi-stable adiposity and bi-stable overgrowth in parallel crosses to C57BL/6J and FVB/N dams, respectively^42,43^. The finding indicates a substantial background genetic interaction with the *NNAT*-buffered UPV axis. These data highlight the challenge of examining and dissecting UPV effects even in mice where genetics and environment can be controlled. So how might plasticity axes be investigated in very large human cohorts? Aside from building dedicated cohorts whose design accounts for both documented and undocumented genetic, environmental and parental modifier effects, possibilities include stratification by, or GWAS of, meta-traits (for example, all tangential Type-A versus Type-B covarying phenotypes); and evaluation of phenotypic associations using ‘surrogate’ polygenic risk scores, for instance composed of variants at or near UPV-type-specific differentially expressed genes.

Based on knockout mouse data, we hypothesize that the cluster 4 metabolic state and the Type-B obesities therein, are developmentally programmed states. Incorporating probabilistic developmental effects into models of phenotypic variation and clinical practice is still very challenging, because we have very little understanding of the regulatory axes or mechanisms that control phenotypic variation (as a trait), how these axes interact to generate complex traits, or how the probabilistic regulatory mechanisms ‘switch’ and canalize development toward altered phenotypic states and/or disease. The finding that *Nnat*^+/-p^ and *Trim28*^+/D9^ triggered effects are independent within the same in utero context is profound. It demonstrates that multiple and distinct regulatory axes exist that act in parallel to canalize UPV outcomes. The observation also provides proof for the existence of multiple independent variation-buffering mechanisms and represents an example of a tri-stable mammalian polyphenism. They set the precedent that mammals have the genomic architecture and physiological networks to canalize at least three distinct and reproducible developmental trajectories within the same gene–environment context. The findings indicate that without assays such as epigenome profiling, major precision medicine efforts will be blind to an entire dimension of phenotypic regulation.

Identifying factors that buffer against phenotypic variation is challenging enough; it is equally difficult to determine how probabilistic (molecular) effects mechanistically alter organism-level physiology (such as obesity). Here, we demonstrate that the *Nnat*^+/-p^-buffered overgrowth UPV is hyperinsulinemia dependent, associated with islet-autonomous β-cell hyperproliferation and that alterations in food intake only occur after overgrowth and obesity (C57BL/6J–FVB F1s) (Fig. 2 and Extended Data Fig. 2). Notably, this type of phenotypic variation was phenocopied in human Type-B UPV. Together, these findings indicate that Type-B UPV may constitute an insulin-dependent form of adiposity. While this conclusion differs from the dogmatic view of obesity-driven hyperinsulinemia, it is consistent with reports that hyperinsulinemia may also cause or exacerbate obesity and the suggestion that its early treatment may prevent complications^42^. In support of this notion, ‘heavy’ Type-B discordant twins and Type-B individuals with obesity show downregulation of *CDKN1C* (Extended Data Fig. 9m,n), a molecular effect possibly linked to congenital hyperinsulinism^68^.

Our data do not rule out additional physiological mechanisms as contributors to the bimodal UPV phenotype in *Nnat*^+/-p^ animals. In association with the Coll group, we previously showed that *Nnat*^+/-p^-triggered obesity (on C57BL/6J) is correlated with food intake^42^, suggesting a potential role of the hypothalamus; however, causality was not tested in those instances, nor was the requirement for hyperinsulinemia. Deep physiological phenotyping in these probabilistic models is warranted though choice of assay should not be decided lightly. Assays such as indirect calorimetry, feeding behavior and activity monitoring, in are experience are very challenging due to both the unpredictability of each individual’s end point and therefore very high numbers required, as well as to notable stress sensitivity that leads to numerous dropout animals such that the experimenter can no longer rule out a biologically biased sub-sampling. Based on experience, we would caution against descriptive or correlative experiments that do establish causality. *Nnat*^+/-p^ animals for instance have proven challenging even for descriptive indirect calorimetry. A considerable number of animals lose weight even under home-cage and acclimatized conditions.

Our work shows that the islet-autonomous hyperproliferative program (in *Nnat*^+/-p^ animals) is HDAC-dependent. This result demonstrates that HDAC modulation has a direct impact on pancreatic β-cell proliferation, which was not previously described. HDAC activity has been previously implicated in the regulation of adipogenesis, pancreas development, β-cell functionality and liver metabolism^69–71^ and HDAC modulation has been proposed as a potential treatment target for metabolic disease^72–74^. From the precision medicine perspective, our data indicate that HDAC modulation may constitute a preventative therapeutic paradigm for patients stratified by Type-B UPV gene expression.

Altogether, this work demonstrates that in addition to genetic and environmental factors, phenotypic outcomes in mammals are defined by probabilistic factors with the potential to canalize multiple distinct, stable and reproducible outcomes. The data indicate that a substantial fraction of human metabolic disease variation (and potentially associated processes such as cancer and inflammation) are defined by such processes.

## Methods

All animal experiments were approved by Institutional Animal Care and Use Committee protocol no. 18-10-028 at VAI, USA and protocol no. MPI-ZH 2016-2019 at MPI, Germany. TwinsUK received ethical approval associated with TwinsUK Biobank (19/NW/0187), TwinsUK (EC04/015) or Healthy Ageing Twin Study (HATS) (07/H0802/84) studies from NHS Research Ethics Committees at the Department of Twin Research and Genetic Epidemiology, King’s College London. All samples and information were collected with written and signed informed consent. The Danish Twins study was approved by the Central Scientific-Ethical Committee of Denmark and was conducted according to the principles of the Helsinki Declaration. Furthermore, approval was obtained from the Danish Data Protection Agency. Informed consent was obtained from all participants. The LCAT study was approved by the University of Leipzig Ethics Committee (265–08, 265–08-ff) and registered in the National Clinical Trials database (NCT02208141). Written informed consent was obtained from all parents.

### *Trim28* and *Nnat* heterozygous mice

The generation of *Trim28*^+/D9^ and *Nnat* heterozygous mice are described elsewhere^30,43,75^. All mice were maintained with four to five siblings under controlled temperature (22 ± 1 °C) and humidity (50 ± 10%) and a 12 h light, 12 h dark schedule. Food and water were available ad libitum unless otherwise stated. All mice were weaned at 3 weeks of age onto a standard chow (V1185-300 MZ-Ereich, ssniff; autoclavable mouse breeder diet 5021, cat. no. 0006540, LabDiet,). Body composition was determined using an EchoMRI 4-in-1 (SYS-ID EF-036).

### Genotyping

Ear biopsies were collected, boiled in 180 μl digestion buffer (50 mM NaOH and 0.1 mM EDTA) at 60 °C overnight. Twenty microliters of neutralization buffer (1 M Tris-HCl, pH 8.0) were added to stop the digestion. Two pairs of primers, one for WT and one for mutant, were mixed separately in two reactions with 1 μl biopsy lysate in 20 μl total volume including DreamTaq DNA polymerase (EP0701, Thermo Fisher) and amplified by PCR for 94 °C for 1.5 min (94 °C for 30 s, 55 °C for 30 s and 68 °C for 1 min) × 31 and 68 °C for 5 min. The primers are listed in Supplementary Table 1. The amplification products of WT (573 bp) and mutant *Nnat* (545 bp) bands, were confirmed on 2% agarose gels. Methods for genotyping the *Trim28*^+/D9^ mouse line are described elsewhere^30,43^.

### Skeleton staining

Mice were killed, the skin and internal organs removed and the eviscerated mice fixed in 95% ethanol overnight and stained with Alcian blue (0.3% in 70% ethanol) for 48 h. The skeletons were incubated with 2% KOH for 48 h and stained with Alizarin red S (0.1% in 95% ethanol) overnight. The stained skeletons were cleared in 1% KOH/20% glycerol solution for up to 1 week and stored in ethanol/glycerol solution (1:1) before imaging.

### Plasma growth factors measurement

Plasma insulin, IGF1/IGF2 and GH were determined by ELISA according to manufacturers’ instructions (10-1247-01, Mercodia; EMIGF1 and EMIGF2, Thermo Fisher; EZRMGH-45K, Millipore Sigma).

### Glucose tolerance test and glucose-stimulated insulin secretion

Following a 6-h fast, mice were administered glucose (1 g kg^−1^) by oral gavage and blood samples for glucose measurement were collected from the tail vein at the indicated times. Glucose levels were measured using a OneTouch Vita glucometer. Blood samples were collected from the cheek at the indicated times into EDTA-coated tubes to prevent coagulation. Blood samples were centrifuged at 850*g* for 20 min at 4 °C to collect plasma. Plasma insulin was measured by ELISA (10-1247-01 or 10-1249-01, Mercodia).

### Mouse islet isolation and spheroid formation

Mice were killed with CO_2_ and dissected according to standard procedures. The common bile duct was tied off with a thread, so perfusion buffer would flow to the pancreas rather than the liver. The pancreas was perfused with 5 ml collagenase digestion buffer (HBSS, 10 mM HEPES and 2 mg ml^−1^ collagenase 4 from Worthington, cat. no. LS004189) through the sphincter of Oddi. The perfused pancreas was placed in 2 ml collagenase digestion buffer and incubated for 30 min at 37 °C. After incubation, the tube was mixed by inversion 30 times. Then, 40 ml of quenching buffer (HBSS, 24 mM HEPES and 5 mg ml^−1^ BSA) was added to stop the collagenase activity. The tube was centrifuged at 20*0g* for 3 min at 4 °C to form a cell/tissue pellet. The pellet was resuspended with 25 ml quenching buffer and run through a 420-µm strainer to remove undigested material. The clarified supernatant was then passed through a 70-µm strainer to collect the islets from the bulk solution. The islets were transferred into a cell culture dish containing RPMI medium (Gibco, 11875093) by inverting the strainer and dipping it in medium. The dish was placed in a 5% CO_2_ incubator at 37 °C overnight. The following day, islets were removed from the culture dish with a P10 pipet and transferred into a fresh RPMI dish. Recovered islets were incubated in a 5% CO_2_ incubator at 37 °C for another 2 days to allow islet metabolism to normalize. Then, 100 µl of islet suspension were mixed with 500 µl warm accutase (A6964-100ML, Sigma) and incubated for 2 min at 37 °C to dissociate islets into single cells. Cells were stained with DAPI and sorted by FACS to recover (DAPI-negative) live cells. Approximately 2,000 live cells were seeded per well in a 96-well spheroid plate (Corning, CLS4520-10EA) and the plate incubated in a 5% CO_2_ incubator for 3 days at 37 °C until spheroids formed.

### Glucose-stimulated insulin secretion ex vivo

Krebs Ringer buffer (140 mM NaCl, 3.6 mM KCl, 0.5 mM NaH_2_PO_4_, 0.2 mM MgSO_4_, 1.5 mM CaCl_2_, 10 mM HEPES and 2 mM NaHCO_3_) and BSA (0.5 %) was prepared freshly on the treatment day and pH adjusted to 7.4. The 2.8 mM glucose and 16.7 mM glucose solutions were prepared with fresh Krebs Ringer buffer. Isolated islets or formed spheroids were transferred into 100 µl of the 2.8 mM glucose solution and incubated at 37 °C for 30 min. Then, the islets or spheroids were transferred into another well with 100 µl 2.8 mM glucose solution and incubated at 37 °C for 1 h for the basal release. Finally, the islets or spheroids were transferred into another well with 100 µl 16.7 mM glucose solution and incubated at 37 °C for 1 h for the glucose-stimulated release.

### Total insulin content

Intact pancreata were weighed, cut into small pieces and incubated with 0.18 M HCl in 70% ethanol overnight at 4 °C. The resulting mixture was transferred to 1.5-ml microfuge tubes and centrifuged at 850*g* for 5 min at room temperature. The clarified supernatant was transferred to a new tube and stored at −20 °C until use. Insulin content was determined via ELISA according to the manufacturer’s instructions (10-1247-01, Mercodia).

### Streptozocin administration and insulin pellet restoration

Five-week-old mice received a one-time i.p. injection of STZ (300 mg kg^−1^, Sigma, S0130-1G). Four days and again 1 month after STZ treatment, insulin pellets (Linplant, Linshin) were s.c. implanted on the back of the animals (one pellet per 20 g body weight) as per the manufacturer’s instructions.

### EdU proliferation

Primary islets in RPMI medium (10% FBS, 50 IU ml^−1^ penicillin, 50 μg ml^−1^ streptomycin, 0.25 μg ml^−1^ amphotericin B and 50 mg ml^−1^ gentamicin) were stained with 10 μM EdU using a fluorescence microscopy protocol kit following the manufacturer’s instructions (iFluor 488, ab219801, Abcam). We used an A1 Plus-RSi laser scanning confocal microscope (Nikon) and z-stack function to capture sequential images of the islets and reconstruct their three-dimensional volume. The total volume of EdU-incorporated cells was then calculated with an ImageJ macro (https://visikol.com/wp-content/uploads/2019/02/Visikol-Measure-Volume-Macro.ijm).

### Islet RNA extraction, library preparation and sequencing

Total RNA was extracted from isolated islets using the QIAGEN RNeasy Micro kit (cat. no. 74004). Libraries were prepared from 10 ng of total RNA. Total RNA material was converted to dsDNA using the SMART-Seq v.4 Ultra Low Input RNA kit for sequencing, v.091817 (Takara Bio). Illumina Nextera DNA Flex kit (Illumina) was used to convert the complementary DNA into Illumina-compatible sequencing libraries. The quality and quantity of the finished libraries were assessed using a combination of Agilent DNA High Sensitivity chip (Agilent Technologies), QuantiFluor dsDNA System (Promega) and Kapa Illumina Library Quantification qPCR assays (Kapa Biosystems). Individually indexed libraries were pooled and 50-bp, paired-end sequencing was performed on an Illumina NovaSeq6000 sequencer using an S1, 100 cycle sequencing kit (Illumina). Each library was sequenced to an average raw depth of 30 M reads. Base calling was conducted by Illumina RTA3 and the output of NextSeq Control Software was demultiplexed and converted to FastQ format with Illumina Bcl2fastq v.1.9.0.

### Mouse transcriptomic analysis

We performed bulk messenger RNA-seq on primary islets and adipocytes taken from *Nnat*^+/-p^*-Light*, *Nnat*^+/-p^-*Heavy* mice and WT littermates. Raw sequences were aligned to mouse reference genome GRCm38.p6 with the snakePipes2 RNA-seq pipeline^76^. Differential expression of the raw counts was performed using DESeq2 (ref. ^77^). GSEA of DEG results was performed with fgsea^78^. Enrichment maps were generated in Cytoscape^79^ from results of 6-week-old *Nnat*^+/-p^*-Light* versus *Nnat*^+/-p^-*Heavy* MSigDB C2 CGP GSEA. We also performed bulk mRNA-seq on primary islets from early-stage (3-week-old) *Nnat*^+/-p^-*Light* and *Nnat*^+/-p^-*Heavy-like* mice and WT littermates. Differential gene expression analysis was performed as described above. Samples were batch corrected using ComBat and normalized count matrices were inspected using PCA. *Nnat*^+/-p^*-Heavy* enriched leading-edge genes from HDAC-related gene sets were assessed at early and late-stage expression profiles to be represented on the heat map. The contribution of the HDAC-signature to the overall gene expression variation was evaluated based on the PCA. The principal components (PCs) from the PCA were ordered for their association to the HDAC-signature (mean of contributions to PCs for the genes belonging to the signature) and the top four correlated were subset (the inflection point of the ordered PCs). The cumulative contribution of these PCs to gene expression variation in the dataset was visualized and compared to the overall contribution from all PCs.

### HDACi and HATi treatment

Islets were isolated from 7–8-week-old animals and cultured ex vivo in RPMI medium (added 10% FBS, 50 IU ml^−1^ penicillin, 50 μg ml^−1^ streptomycin, 0.25 μg ml^−1^ amphotericin B and 50 mg ml^−1^ gentamicin) for 2 days to reach steady state. Islets were then treated with 500 nM HDACi (TSA) or 500 mM HATi (C646) for 1 day, followed by a 2-d EdU incubation (iFluor 488, Abcam) to track DNA replication.

### Immunochemistry and H&E staining

Insulin (A0564, DAKO, 1× ready to use) and H&E staining were performed on sequential, 5-μm paraffin thin sections fixed in 4% phosphate-buffered formalin. Insulin immunohistochemistry was performed on an Agilent Autostainer Link 48 instrument as per the manufacturer’s instructions. Stained thin sections were digitized with an Aperio ScanScope slide scanner fitted with a ×20 objective (Leica). For each animal, we imaged three insulin and three H&E thin sections. Positively stained β-cells in each thin section were quantified with Genie (Leica) software. Reported β-cell-positive areas are the average from four animals per group and three thin sections per animal (*n* = 12 thin sections). We quantified apoptosis events by using the TUNEL assay kit, HRP-DAB (ab206386, Abcam) on pancreatic sections from 16-week-old animals.

### Immunofluorescence

Paraffinized sections were heated, deparaffinized with xylene and rinsed in water. Antigen retrieval was performed by heating the slides at 95 °C for 20 min in Tris-EDTA, pH 9.0. Specimens were blocked in 5% goat serum PBST (0.05% Tween 20) and incubated overnight with insulin primary antibody (A0564, DAKO; 1:50 dilution) and Ki-67 primary antibody (ab15580, Abcam; 1:200 dilution); glucagon primary antibody (G2654, Sigma; 1:500 dilution); and somatostatin primary antibody (ab30788, Abcam; 1:200 dilution). Fluorochrome-conjugated secondary antibodies (Alexa Fluor 488, anti-rabbit; Alexa Fluor 555, anti-guinea pig; Alexa Fluor 647, anti-rat; Alexa Fluor 488, anti-mouse; 1:500 dilution, Invitrogen) were then added to each slide and incubated for 2 h at room temperature. Slides were rinsed three times for 10 min each in PBST buffer and air dried. A drop of VectaShield mounting medium (containing DAPI; H-1200, Vector Laboratories) and coverslip were applied to each slide and slides cured overnight at 4 °C in the dark before image acquisition. Images were acquired using an A1 Plus-RSi laser scanning confocal microscope (Nikon).

### qRT–PCR

Total RNA was extracted using TRI Reagent (Sigma) and reverse transcribed into cDNA using a commercially available kit (43-688-14, Applied Biosystems). *Nnat* and *Hprt* transcripts were quantified using TaqMan gene expression assays with validated probes (Life Technologies). All probes are listed in Supplementary Table 1. All qPCR reactions were performed on a 7900HT Fast Real-Time PCR System (Applied Biosystems). Thermal cycling conditions for all genes included 2 min at 50 °C, 20 s at 95 °C and 40 cycles of 95 °C for 1 s, 60 °C for 20 s. Post-amplification melting curve analysis was performed to check for nonspecific products and probe-only controls were included as negative controls. Threshold cycles (Ct values) were normalized to *Hprt* within each sample to obtain sample-specific ΔCt values (Ct gene of interest − Ct housekeeping gene). The 2^-ΔΔCt^ values were calculated to obtain fold expression levels, where ΔΔCt = (ΔCt treatment − ΔCt control).

### Human population studies and analyses

#### MuTHER TwinsUK cohort

The MuTHER cohort consists of 855 female white twins and 193 MZ co-twin pairs, aged between 40 and 87 years^56,57^ and is a subset of the larger TwinsUK study^55^ (referred to as TwinsUK in the figures and text). Subcutaneous adipose tissue samples were obtained from skin punch biopsies. Gene expression profiles were generated using Illumina’s whole genome expression array (HumanHT-12 v.3) and are available from the ArrayExpress archive under the repository no. E-MTAB-1140. For expression arrays, the original authors^57^ used multiple technical replicates for each sample, which were all randomized before hybridization and replicates run on different BeadChips. Expression signals were normalized separately per tissue, with quantile normalization of the replicates of an individual followed by quantile normalization across all individuals. The authors acknowledged that their approach does not adjust for shared covariance due to technical factors that may influence subsequent analysis, but previous efforts indicate that the impact on the result seemed to be minor^80^. Expression data were corrected for technical batch effect using ComBat^81^ and distributions of identified UPVs and individuals’ clusters among batches did not show specific enrichment. Differential expression analysis and GSEA were performed using limma^82^ and fgsea^78^, respectively. The differential expression analysis was performed using age as a covariate in the model. Cell-type deconvolution was performed using CibersortX^66^ and a recently published single-cell atlas of human white adipose tissue^67^ was used as a signatures reference.

#### Monozygotic co-twin analysis

To maximize the number of co-twin couples in the analyses (*n* = 153), we excluded the waist, hip and waist-to-hip ratio measurements, which were not available for ~one-third of the cohort. Discordance indices were calculated as the difference of the log-transformed values between co-twins for each measurement, after ordering the co-twins according to their BMI. Likewise, we calculated gene expression discordance from normalized expression array counts. We used a graph-based clustering approach from Seurat^58^ for unbiased clustering of co-twin pairs according to their morphometric discordances. Heat maps of morphometric discordances were generated with ‘pheatmap’ (https://cran.r-project.org/web/packages/pheatmap/index.html) by clustering discordances based on Euclidean distances, a complete agglomeration method and rows scaling. Correlations between gene expression and phenotypic discordances were determined by Spearman correlation and reported as the −log_10_(*P*). When performing the same analysis on the identified co-twin clusters, the average of *P* values from all Spearman correlations for a single gene was reported.

#### Danish Twins cohort

The Danish Twins cohort used in this study consists of 160 elderly individuals (88 females and 72 males), aged between 63 and 83 years. The cohort includes 20 MZ and 21 DZ same-sex co-twin pairs and is part of a larger study^61^. RNA samples were obtained from subcutaneous adipose tissue biopsies and *NNAT* expression was measured by qRT–PCR analysis, as previously reported^43^. The MZ co-twin pairs were divided into halves according to their average *NNAT* expression level. Like the TwinsUK cohort, we calculated serum insulin and BMI discordance among MZ co-twins (as log_2_(fold change) and difference, respectively). Gaussian finite mixture modeling from the Mclust tool^83^ was used to separate insulin-concordant and -discordant co-twin pairs. The proportions of insulin-concordant and -discordant co-twins among *NNAT*-low- and *NNAT*-high-expressing couples were visualized. The distributions of BMI discordances among *NNAT*-low- and *NNAT*-high-expressing couples were compared for homogeneity of variances using the Bartlett’s test.

#### DNA methylation analysis in the MuTHER TwinsUK cohort

The MuTHER cohort contains Infinium HumanMethylation450 BeadChips array (Illumina WG-314-1002) data from the subcutaneous fat derived of 648 TwinsUK participants. For DNA methylation arrays, the original authors reported^84^ that tissue samples were randomized before DNA extraction. Signal intensities were quantile normalized. Beadchip, bisulfite-sequencing (BS) conversion efficiency (assessed with the built-in BS conversion efficiency controls) and BS-treated DNA inputs were shown to contribute significantly to the variation in β levels and were included as covariates in subsequent analysis. The processed and normalized β values were previously published^57,84,85^ and are available from the ArrayExpress archive under the repository no. E-MTAB-1866. We analyzed the data using the ‘SeSAMe’ pipeline^86,87^. Normalized β values were analyzed by linear modeling to identify DMRs between ‘light’ and ‘heavy’ MZ co-twins, controlling for age as a covariate in the model. The cutoff used to define differential methylation was FP < 0.05 and effect size threshold >0.05 (DNA methylation differences under 5% were not considered biologically meaningful). The heat map of the differentially methylated CpGs between co-twin pairs belonging to the four different phenotypic variation clusters was generated with ‘ComplexHeatmap’^88^. The genomic regions of DMRs from the Type-B UPV co-twins were used to search for genome-wide relevant associations between SNPs and phenotypes in the T2D Knowledge Portal (https://t2d.hugeamp.org). When DMRs were defined by just a single nucleotide, we searched in ±50-kb regions. All genome-wide significant associations were reported (*P* = 10^−8^). We also visualized all the GWAS associations within our DMRs with a *P* < 10^−3^.

#### Genotyping data analysis in the MuTHER TwinsUK cohort

The MuTHER cohort contains genotyping data generated by Illumina 317 K, 610 k and 1 M chip arrays, from the subcutaneous fat derived of 807 TwinsUK participants. These data are available upon request at the TwinsUK consortium (https://twinsuk.ac.uk/resources-for-researchers/our-data/). The genotype annotation files were generated using IMUTE2 with 1000 Genomes Project phase 1 (interim) as a reference panel. This dataset is based on a sequence data freeze from 23 Nov 2010; the phased haplotypes were released Jun 2011. GWAS data were ‘pre-phased’ using IMPUTE2 without a reference panel. The resulting haplotypes were used to perform fast imputation from the 1000 Genomes Project phase 1 dataset. The imputation of TwinsUK1 (317 K chip) and TwinsUK23 (610 k and 1 M chips) were conducted separately and merged with GTOOL. Genotyping data were analyzed using PLINK software (v.1.9). Samples with a missing call rate exceeding 0.02 (–mind 0.02) were excluded from genetic analysis. One-way ANOVA was conducted to determine whether overall missingness was significantly different across UPV groups at the genome-wide level, without finding statistical relevant differences. After removal of overly missing samples, each co-twin pair was screened for discrepancies in genotypes to assess the extent of co-twin genetic similarity, which accounted for >99.9% of the data. These analyses were conducted in R (v.4.1.1) using the ‘stats’ package.

#### Whole MuTHER TwinsUK cohort analysis

To generalize the findings from MZ co-twins, we included all individuals from the MuTHER cohort and analyzed 824 gene expression profiles from s.c. adipose tissues. The Type-B UPV gene expression signature was identified by performing differential gene expression analysis between ‘heavy’ and ‘light’ co-twins from the four concordant/discordant clusters. We selected all the genes with a *P* value <0.001 and clustered them by *k*-means clustering to identify the Type-B specific signature (127 total genes). We then used the signature to stratify the TwinsUK individuals. The number of clusters for this analysis was determined by visualizing the dispersion within each cluster for *k* = 1–10 and selecting the number of clusters that represented the ‘saturation point’ of dispersion. Cluster stability was assessed by the Jaccard’s similarity score. These analyses were performed using the RaceID package^89^. The heat map of the four *k*-means individuals’ clusters was generated with ‘ComplexHeatmap’^88^. Gene clustering was based on Euclidean distances and Ward’s agglomeration method of log-transformed and scaled, normalized data. The individuals were further ordered by BMI within each group. We then ranked Type-B-specific signatures (127 genes) for each individual. High ranks were associated with a ‘heavy-like’ Type-B transcriptional profile. The median of ranks from all UPV-B genes was plotted for each individual. The contribution of the Type-B-specific gene signature to overall gene expression variation in the cohort was evaluated as follows. PCA was performed on the gene expression profiles of all individuals. The 824 PCs were ordered for their association to the Type-B gene expression signature (mean of contributions to PCs of the genes belonging to the 127-gene signature) and the top 25 correlated components were subset (inflection point of the ordered PCs). The cumulative contribution of the top 25 PCs to gene expression variation in the cohort was visualized and compared to the overall contribution from all 824 PCs. The HDAC-signature annotation reported in the heat map was derived as follows: first, we performed GSEA of HDAC-related gene sets between the ‘heavy’ and ‘light’ co-twins belonging to the Type-B phenotypic cluster. Next, we retrieved the leading-edge genes (genes driving the gene sets’ enrichment), from the ‘heavy’-enriched HDAC-related gene sets, which then defined the HDAC-signature. Annotations in the heat map show the average expression of the HDAC-signature genes for each individual. The unbiased transcriptional analysis among the four individuals’ clusters was based on the top 1,000 most variable genes among all the samples (heat map shows only the genes for which we can detect expression in the LCAT cohort). GO and pathway analysis were performed with ‘clusterProfiler’^90^ against the GO, KEGG and MSigDB databases. For the metabolic/morphologic characterization of the individuals’ and obesity clusters, the serum insulin levels were adjusted on BMI and the fat and lean mass normalized on the squared height, generating FatMI and LeanMI, respectively.

#### The LCAT cohort

The LCAT cohort consists of female and male white children aged 0–18 years who underwent elective orthopedic surgery, herniotomy/orchidopexy or other surgeries^65^. Exclusion criteria were severe diseases and medication that might affect adipose tissue biology, such as diabetes, generalized inflammation, malignant diseases, genetic syndromes or permanent immobilization. BMI data were standardized to age- and sex-specific centiles by applying German reference data and are represented as BMI SDS^91^. Overweight and obesity are defined by a cutoff of 1.28 and 1.88 SDS (90th or 97th centile), respectively. Subcutaneous adipose tissue samples were excised during surgery, washed three times in PBS and immediately frozen in liquid nitrogen for RNA isolation. For RNA-seq, gene expression profiles were generated as previously described^43^. Differential expression and GSEA were performed using DESeq2 (ref. ^77^) and fgsea^78^, respectively. Normalized counts were corrected for both age and sex confounders with ComBat^81^. For global gene expression analysis on individuals, 61 profiles from s.c. adipose tissues were analyzed (34 males and 27 females). The number of clusters selection and the heat map generation were performed as in the adult cohort. The heat map in Fig. 5 shows the Type-B specific gene clustering (top) and the same variable genes as in the TwinsUK cohort (bottom). For the metabolic/morphometric characterization of the individuals’ and obesity clusters, standardized (SDS) measurements and fasting serum insulin levels normalized on BMI SDS were visualized.

### Statistics

In both human and mice analyses, equality of variances and means were assessed by Levene’s test and Student’s *t*-tests, respectively (unless otherwise specified). We used one-way ANOVA followed by Tukey’s honestly significant difference test (where appropriate and as indicated) for multiple comparison testing. In the mice body composition data analysis, the separation into discrete clusters was tested by Gaussian finite mixture modeling using the Mclust tool^83^. A supervised analysis was performed to identify the best model describing the WT as a single reference cluster. Next, the same model was applied on data from other genotypes. All data are expressed as mean ± s.e.m., unless otherwise specified. Correlations were tested by linear regression, unless otherwise specified. All reported *P* values are two-tailed, unless stated otherwise, where *P* ≤ 0.05 was considered to indicate statistical significance. Calculations to evaluate the power to detect an effect given the sample size in mouse studies, were performed with bifurcatoR (https://github.com/VanAndelInstitute/bifurcatoR). Mouse studies were designed to reach 95% power to detect effect size.

### Reporting summary

Further information on research design is available in the Nature Research Reporting Summary linked to this article.

## Supplementary information

Supplementary Information Supplementary Tables 1 and 2

Reporting Summary

## Data Availability

RNA-seq data from both mouse primary islets and subcutaneous adipose tissue of the LCAT cohort, have been deposited to Gene Expression Omnibus and are publicly available under the accession codes GSE205740 and GSE205668, respectively. They are collected under the GSE205741 super-series. Gene expression and DNA methylation profiles by whole genome arrays from subcutaneous adipose tissue of the MuTHER TwinsUK cohort have been deposited to Array Express and are publicly available under the accession codes E-TABM-1140 and E-TABM-1866, respectively. Morphometric and genotypic data of the MuTHER TwinsUK cohort are available upon request at https://twinsuk.ac.uk/resources-for-researchers/access-our-data/. The MSigDB is available at http://www.gsea-msigdb.org/gsea/msigdb. Source data are provided with this paper.

## Source data

Source Data Fig. 1 Statistical Source Data.
Source Data Fig. 2 Statistical Source Data.
Source Data Fig. 2 Unprocessed and uncropped images.
Source Data Fig. 3 Statistical Source Data.
Source Data Fig. 4 Statistical Source Data.
Source Data Fig. 5 Statistical Source Data.
Source Data Extended Data Fig. 1 Statistical Source Data.
Source Data Extended Data Fig. 1 Unprocessed and uncropped gel.
Source Data Extended Data Fig. 2 Statistical Source Data.
Source Data Extended Data Fig. 3 Statistical Source Data.
Source Data Extended Data Fig. 3 Unprocessed and uncropped images.
Source Data Extended Data Fig. 4 Statistical Source Data.
Source Data Extended Data Fig. 5 Statistical Source Data.
Source Data Extended Data Fig. 7 Statistical Source Data.
Source Data Extended Data Fig. 8 Statistical Source Data.
Source Data Extended Data Fig. 9 Statistical Source Data.

## Code availability

No custom code or mathematical algorithm were generated in this report. All publicly available codes and tools used to analyze the data are reported and referenced in the Methods and the Reporting Summary.
