# Supplementary material for: Independent phenotypic plasticity axes define distinct obesity sub-types
Source: Nat Metab. 2022 Sep 12;4(9):1150–65. doi: 10.1038/s42255-022-00629-2 (PMC9499872; doi:10.1038/s42255-022-00629-2)
Supplement: Supplementary file 1 — Supplementary Tables 1 and 2 [file 42255_2022_629_MOESM1_ESM.pdf]

nature portfolio

https://doi.org/10.1038/s42255-022-00629-2

Supplementary information

Independent phenotypic plasticity axes define distinct obesity sub-types

In the format provided by the authors and unedited

Supplementary Information

Supplementary Table 1 | Primers for genotyping

Gene	Forward Primer (5’-3’)	Reverse Primer (5’-3’)
Nnat WT	ACACTTGGGTGGGTGAAAAAGA	GGAGGATTTCGAAAAGCGAATC
Nnat KO	CGGTCGCTACCATTACCAGT	CCTGGGTAGAGAGATCTGTGG

Supplementary Table 2 | Mouse qPCR probes

Gene	Species	Probe ID*
Nnat	Mouse	Mm00440480_m1
Hprt	Mouse	Mm00446968_m1

* All primers and probes were manufactured by Life Technologies. Probes were labelled with either 5'-FAM or 5'HEX as the reporter, and 3'-TAMRA as the quencher.
