# Supplementary material for: Independent phenotypic plasticity axes define distinct obesity sub-types
Source: Nat Metab. 2022 Sep 12;4(9):1150–65. doi: 10.1038/s42255-022-00629-2 (PMC9499872; doi:10.1038/s42255-022-00629-2)
Supplement: Supplementary file 2 — Reporting Summary [file 42255_2022_629_MOESM2_ESM.pdf]

nature portfolio

Corresponding author(s): J. Andrew Pospisilik

Last updated by author(s): Jul 18, 2022

Reporting Summary

Nature Portfolio wishes to improve the reproducibility of the work that we publish. This form provides structure for consistency and transparency in reporting. For further information on Nature Portfolio policies, see our Editorial Policies and the Editorial Policy Checklist.

Statistics

For all statistical analyses, confirm that the following items are present in the figure legend, table legend, main text, or Methods section.

n/a	Confirmed	
☐	☒	The exact sample size (*n*) for each experimental group/condition, given as a discrete number and unit of measurement
☐	☒	A statement on whether measurements were taken from distinct samples or whether the same sample was measured repeatedly
☐	☒	The statistical test(s) used AND whether they are one- or two-sided<br>*Only common tests should be described solely by name; describe more complex techniques in the Methods section.*
☐	☒	A description of all covariates tested
☐	☒	A description of any assumptions or corrections, such as tests of normality and adjustment for multiple comparisons
☐	☒	A full description of the statistical parameters including central tendency (e.g. means) or other basic estimates (e.g. regression coefficient) AND variation (e.g. standard deviation) or associated estimates of uncertainty (e.g. confidence intervals)
☐	☒	For null hypothesis testing, the test statistic (e.g. *F*, *t*, *r*) with confidence intervals, effect sizes, degrees of freedom and *P* value noted<br>*Give P values as exact values whenever suitable.*
☐	☒	For Bayesian analysis, information on the choice of priors and Markov chain Monte Carlo settings
☐	☒	For hierarchical and complex designs, identification of the appropriate level for tests and full reporting of outcomes
☒	☐	Estimates of effect sizes (e.g. Cohen's *d*, Pearson's *r*), indicating how they were calculated

Our web collection on statistics for biologists contains articles on many of the points above.

Software and code

Policy information about availability of computer code

Data collection	Illumina RTA3 v3 - RNAseq base calling<br>Illumina Bcl2fastq v1.9.0 - RNAseq fastq generation
Data analysis	snakePipes v2.5.3 - RNAseq QC and mapping<br>DESeq2 v1.36.0 - RNAseq analysis<br>limma v3.52.2 - expression array analysis<br>fgesa v1.22.0 - gene set enrichment analysis (GSEA)<br>Cytoscape v3.8.2 - visualization of GSEA results<br>Combat (sva) v3.38.0 - batch effect removal<br>pheatmap v1.0.12 - heatmap visualizations<br>ComplexHeatmap v2.13.0- heatmap visualizations<br>Seurat v3.2.0- clustering<br>Mclust v5.4.10- Gaussian finite mixture modeling<br>SeSAMe v1.14.2 - DNA methylation analysis<br>PLINK v1.9 - genotypic data analysis<br>RaceID v0.2.3 - k-means clustering evaluation<br>clusterProfiler v4.4.4 - gene ontology (GO) analysis<br>CibersortX - cell type deconvolution<br>stats v4.3.0 - statistic analysis R package

For manuscripts utilizing custom algorithms or software that are central to the research but not yet described in published literature, software must be made available to editors and reviewers. We strongly encourage code deposition in a community repository (e.g. GitHub). See the Nature Portfolio guidelines for submitting code & software for further information.

Data

Policy information about availability of data

All manuscripts must include a data availability statement. This statement should provide the following information, where applicable:

- Accession codes, unique identifiers, or web links for publicly available datasets
- A description of any restrictions on data availability
- For clinical datasets or third party data, please ensure that the statement adheres to our policy

Data availability
RNA-seq data from both mouse primary islets and subcutaneous adipose tissue of the LCAT cohort, have been deposited to Gene Expression Omnibus (GEO) and are publicly available under the accession codes GSE205740 and GSE205668, respectively. They are collected under the GSE205741 super-series. Gene expression and DNA methylation profiles by whole genome arrays from subcutaneous adipose tissue of the MuTHER-TwinsUK cohort have been deposited to Array Express and are publicly available under the accession codes E-TABM-1140 (https://www.ebi.ac.uk/arrayexpress/experiments/E-TABM-1140/) and E-TABM-1866 (https://www.ebi.ac.uk/arrayexpress/experiments/E-MTAB-1866/), respectively. Morphometric and genotypic data of the MuTHER-TwinsUK cohort are available upon request at https://twinsuk.ac.uk/resources-for-researchers/access-our-data/. The Molecular Signatures Database (MSigDB) is available at http://www.gsea-msigdb.org/gsea/msigdb.

Field-specific reporting

Please select the one below that is the best fit for your research. If you are not sure, read the appropriate sections before making your selection.

☒ Life sciences ☐ Behavioural & social sciences ☐ Ecological, evolutionary & environmental sciences

For a reference copy of the document with all sections, see nature.com/documents/nr-reporting-summary-flat.pdf

Life sciences study design

All studies must disclose on these points even when the disclosure is negative.

Sample size	The sample size of our animal experiments was determined by power calculation. Our experiments were designed to have >95% power to detect phenotypic variation (i.e. identify bi-modal distributions) and are validated across mouse houses. Our power calculation is based on Hartigan's dip test performed on 1000 simulations of bimodal data assuming each mode is roughly Gaussian, and with each mode scaled by its variance. A post-hoc analysis showed we have >99% power at 16 weeks of age.<br>Sex was considered in the study design, and all the findings were recapitulated both in male and female animals, and reported in distinct panels.<br>For human studies, no sample size calculations were performed prior to data analysis. All available samples were utilized to maximize statistical power and clustering accuracy. We referred to recent reference (PMID: 35641905) to ensure sufficient sample sizes for clustering, where a minimum of N=20 samples per group is recommended, and 80% classification accuracy is achieved for N=80 at modestly small cluster separations. Sex, age and ethnicity were considered during the analyses, and we checked that these covariates were not driving the clustering.
Data exclusions	To minimize litter size effects (variation attributable to differences in in utero / early life sufficiency), we used offspring from litters of 9–12 pups and tightly controlled husbandry, environment, and housing density. This exclusion criteria was pre-established.<br>No data exclusion criteria were used in human studies.
Replication	We tested and observed phenotypic bi-stability in two independent mouse houses, and after surviving rederivations independently from cryopreserved embryos and sperm. For all other experiments, at least 3 independent biological replicates were performed. All replication attempts were successful. All attempts at replication are included in the data.
Randomization	Mice are sex- and littermate- matched and were randomly assigned to treatment and control groups.<br>In human studies, all samples randomized during isolation and library preparation. During analyses, groups allocation was achieved by graph-based or k-means clustering, and controlled for covariates, including sex, age, ethnicity and technical confounders, as reported in the methods section of the paper.
Blinding	All mouse phenotypic data were collected by the researcher without knowing the genotypes. Data analysis were performed by researchers blinded to group/phenotye information. In both mouse and human analyses, all samples were de-identified during isolation and library preparation. Investigators were blinded to group allocation during data collections.

Reporting for specific materials, systems and methods

We require information from authors about some types of materials, experimental systems and methods used in many studies. Here, indicate whether each material, system or method listed is relevant to your study. If you are not sure if a list item applies to your research, read the appropriate section before selecting a response.

Materials & experimental systems

n/a	Involved in the study
☐	☒ Antibodies
☒	☐ Eukaryotic cell lines
☒	☐ Palaeontology and archaeology
☐	☒ Animals and other organisms
☐	☒ Human research participants
☒	☐ Clinical data
☒	☐ Dual use research of concern

Methods

n/a	Involved in the study
☒	☐ ChIP-seq
☒	☐ Flow cytometry
☒	☐ MRI-based neuroimaging

Antibodies

Antibodies used

Insulin ELISA (10-1247-01, Mercodia),
IGF1 and IGF2 ELISA (EMIGF1 and EMIGF2, thermoFisher),
growth hormone ELISA (EZRMGH-45K, Millipore Sigma),
Insulin (A0564, DAKO,1:100), Ki-67 (ab15580, Abcam,1:500),
glucagon (G2654, Sigma,1:500), somatostatin (ab30788, Abcam,1:500),
Fluorochrome-conjugated secondary antibodies (Alexa Fluor 488, anti-Rabbit; Alexa Fluor 555, anti-Guinea pig; Alexa Fluor 647, anti-Rat; Alexa Fluor 488, anti-mouse, Invitrogen,1:500) .

Validation

The kits and antibodies used in the study are validated via manufacturers and the related information are available on the website:
Insulin ELISA (10-1247-01, Mercodia), https://www.mercodia.com/product/mouse-insulin-elisa/

IGF1 ELISA (EMIGF1, thermoFisher), https://assets.thermofisher.com/TFS-Assets/LSG/manuals/EMIGF1.pdf

IGF2 ELISA (EMIGF2, thermoFisher), https://assets.thermofisher.com/TFS-Assets/LSG/manuals/EMIGF2.pdf

Growth hormone ELISA (EZRMGH-45K, Millipore Sigma), https://www.emdmillipore.com/US/en/product/Rat-Mouse-Growth-Hormone-ELISA,MM_NF-EZRMGH-45K#anchor_PR

Insulin (A0564, DAKO), https://www.labome.com/product/Dako/A0564.html

Ki-67 (ab15580, Abcam), https://www.abcam.com/ki67-antibody-ab15580.html

Glucagon (G2654, Sigma), https://www.sigmaaldrich.com/US/en/product/sigma/g2654

Somatostatin (ab30788, Abcam), https://www.abcam.com/somatostatin-antibody-m09204-ab30788.html

Alexa Fluor 488, anti-Rabbit (A27034, Invitrogen), https://www.thermofisher.com/antibody/product/Goat-anti-Rabbit-IgG-H-L-Secondary-Antibody-Recombinant-Polyclonal/A27034

Alexa Fluor 555, anti-Guinea pig(A-21435, Invitrogen), https://www.thermofisher.com/antibody/product/Goat-anti-Guinea-Pig-IgG-H-L-Highly-Cross-Adsorbed-Secondary-Antibody-Polyclonal/A-21435

Alexa Fluor 647, anti-Rat(A-21247, Invitrogen), https://www.thermofisher.com/antibody/product/Goat-anti-Rat-IgG-H-L-Cross-Adsorbed-Secondary-Antibody-Polyclonal/A-21247

Alexa Fluor 488, anti-mouse, (A-11001, Invitrogen), https://www.thermofisher.com/antibody/product/Goat-anti-Mouse-IgG-H-L-Cross-Adsorbed-Secondary-Antibody-Polyclonal/A-11001

Animals and other organisms

Policy information about studies involving animals; ARRIVE guidelines recommended for reporting animal research

Laboratory animals

B6.Nnat+/-p - B6 congenic Nnat knockout mice, both males and females. Data collected from day 0 to 16 weeks.
FVBN/J wild type mice, both males and females. Data collected from day 0 to 16 weeks.
FVB.Trim28+/D9 - Trim28 haploinsufficient mice, both males and females. Data collected from day 0 to 16 weeks.

Wild animals

no wild animals were used in this study

Field-collected samples

no field-collected samples were used in this study

Ethics oversight

All animal experiments were approved by Institutional Animal Care and Use Committee (IACUC) protocol number: 18-10-028 at VAI, U.S.A., and protocol number: MPI-ZH 2016-2019 at MPI, Germany.

Note that full information on the approval of the study protocol must also be provided in the manuscript.

Human research participants

Policy information about studies involving human research participants

Population characteristics	- The MuTHER-TwinsUK cohort was previously described and data used in this manuscript were previously published and available (Refs. PMID: 21304890,22941192,17254428,24183450,26699896). The MuTHER cohort comprises 855 female caucasian twins and 193 MZ cotwin pairs, aged between 40 and 87 years, and it is a subset of the larger Twins UK study. Expression data were corrected for technical batch effect using 'comBat'. The differential expression analysis was performed using age as a covariate in the model. For DNA methylation arrays, beadchip, bisulphite-sequencing (BS) conversion efficiency (assessed with the built-in BS conversion efficiency controls), and BS-treated DNA input were shown to contribute significantly to the variation in beta levels and were included as covariates in subsequent analysis. Differentially methylated sites and regions (DMRs) analyses were controlled for age as a co-variate in the model.<br><br>- The Leipzig Childhood Adipose Tissue (LCAT) cohort was previously described and data used in this manuscript were previously published and available (Refs. PMID:25392242,23181778,28542631,26824653). The Leipzig Childhood adipose tissue (AT) cohort comprises female and male Caucasian children aged 0-18 years. We used 61 transcription profiles (34 males and 27 females). BMI data were standardized to age- and sex- specific centiles by applying German reference data, and are represented as BMI standard score (SDS). Normalized gene expression counts were corrected for both age and sex covariates with 'comBat'.<br><br>- The Danish twins' cohort was previously described and data used in this manuscript were previously published and available (Refs. PMID: 19336677, 26824653, 10064092, 7672901). The cohort used in this study comprises 160 elderly individuals (88 females and 72 males), aged between 63 and 83 years. The cohort includes 20 MZ and 21 DZ same-sex cotwin pairs, and is part of a larger study.
Recruitment	- MuTHER-TwinsUK: All samples and information were collected with written and signed informed consent. All individuals recruited in this study were Caucasian female. Refs. PMID: 21304890,22941192,17254428,24183450,26699896<br><br>- LCAT cohort: Written informed consent was obtained from both parents and from children >12 years. Refs. PMID: 25392242, 23181778, 28542631,26824653<br><br>- The Danish twins' cohort: Informed consent was obtained from all participants. Refs. PMID: 19336677, 26824653, 10064092, 7672901
Ethics oversight	- MuTHER-TwinsUK: The project has been approved by the local ethics committees of all institutions involved. All wave of TwinsUK have received ethical approval associated with TwinsUK Biobank (19/NW/0187), TwinsUK (EC04/015) or Healthy Ageing Twin Study (H.A.T.S) (07/H0802/84) studies from NHS Research Ethics Committees at the Department of Twin Research and Genetic Epidemiology, King's College London. The TwinsUK Resource Executive Committee (TREC) oversees management, data sharing and collaborations involving the TwinsUK registry. Refs. PMID: 21304890, 22941192, 17254428, 24183450,26699896.<br><br>- LCAT cohort: study protocols were approved by the University of Leipzig Ethics Committee (265–08, 265– 08-ff) and registered in the National Clinical Trials database (NCT02208141). Refs. PMID: 25392242, 23181778, 28542631, 26824653<br><br>- The Danish twins' cohort: this study was evaluated and approved by the regional ethics committees (the Central Scientific-Ethical Committee of Denmark), and was conducted according to the principles of the Helsinki Declaration. Furthermore, approval was obtained from the Danish Data Protection Agency. Refs. PMID: 19336677, 26824653, 10064092, 7672901

Note that full information on the approval of the study protocol must also be provided in the manuscript.
