# Supplementary figures and images for: Independent phenotypic plasticity axes define distinct obesity sub-types
Source: Nat Metab. 2022 Sep 12;4(9):1150–65. doi: 10.1038/s42255-022-00629-2 (PMC9499872; doi:10.1038/s42255-022-00629-2)

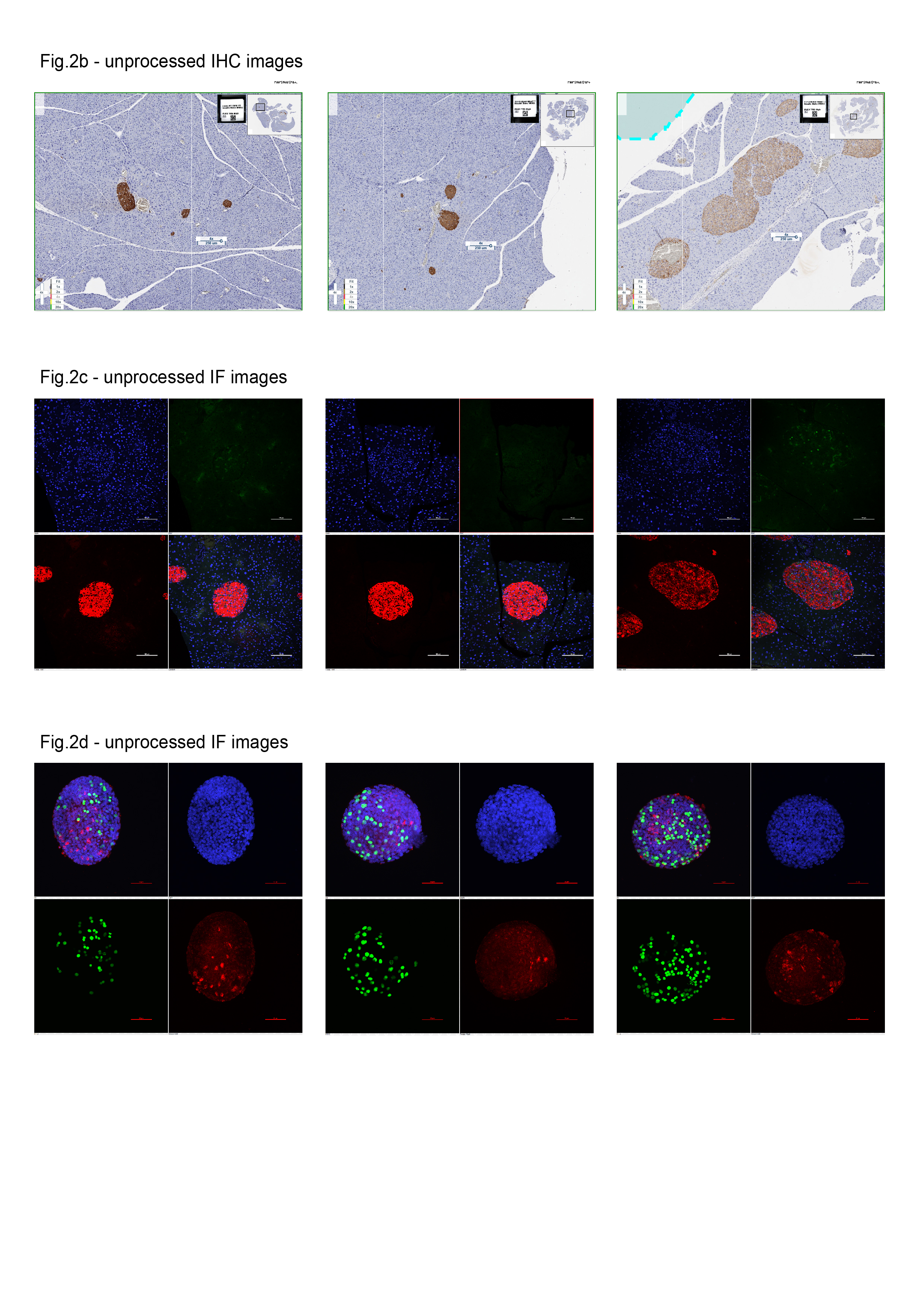

Supplement: Source Data Fig. 2 — Unprocessed and uncropped images. [file 42255_2022_629_MOESM5_ESM.jpg]

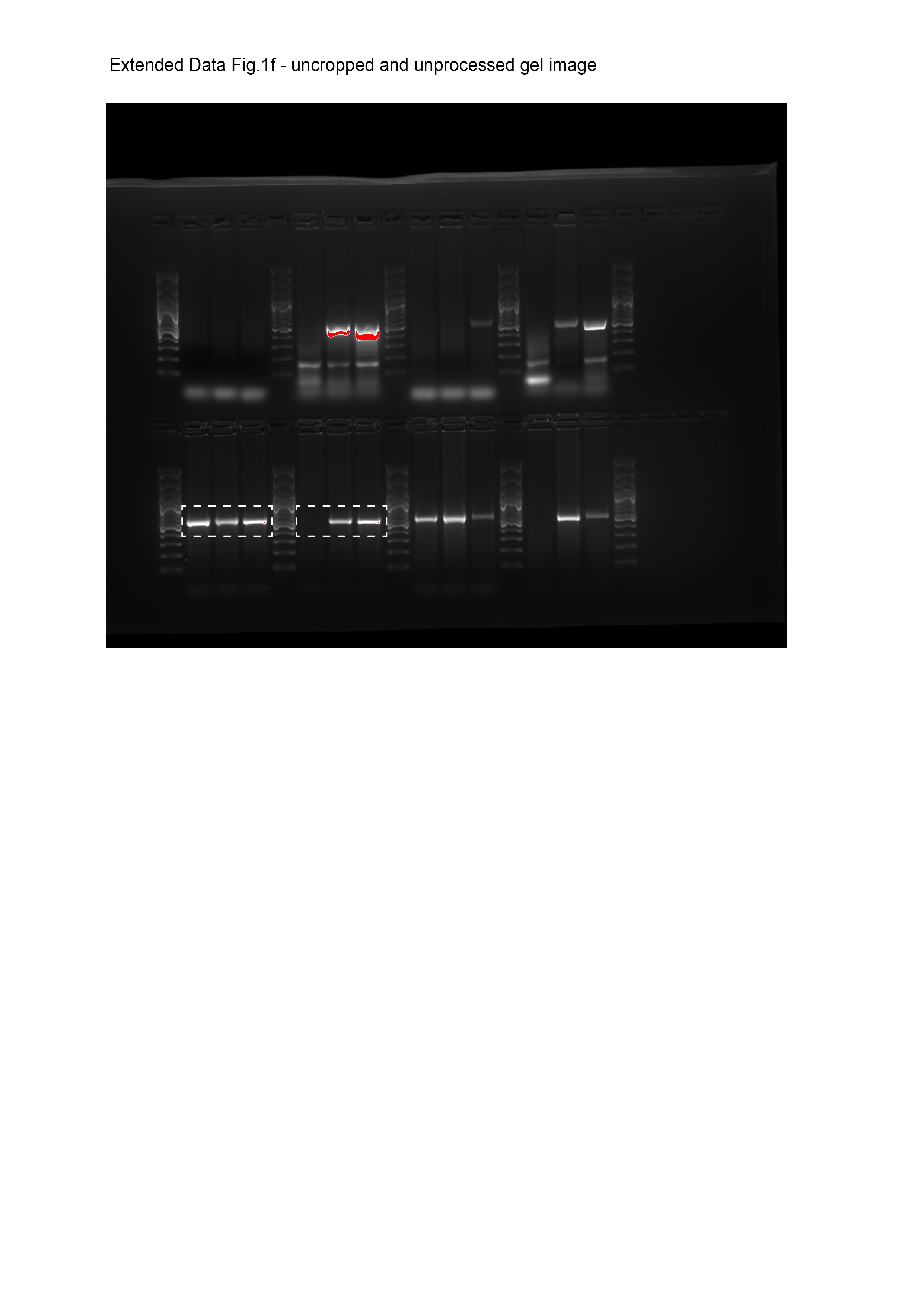

Supplement: Source Data Extended Data Fig. 1 — Unprocessed and uncropped gel. [file 42255_2022_629_MOESM10_ESM.jpg]

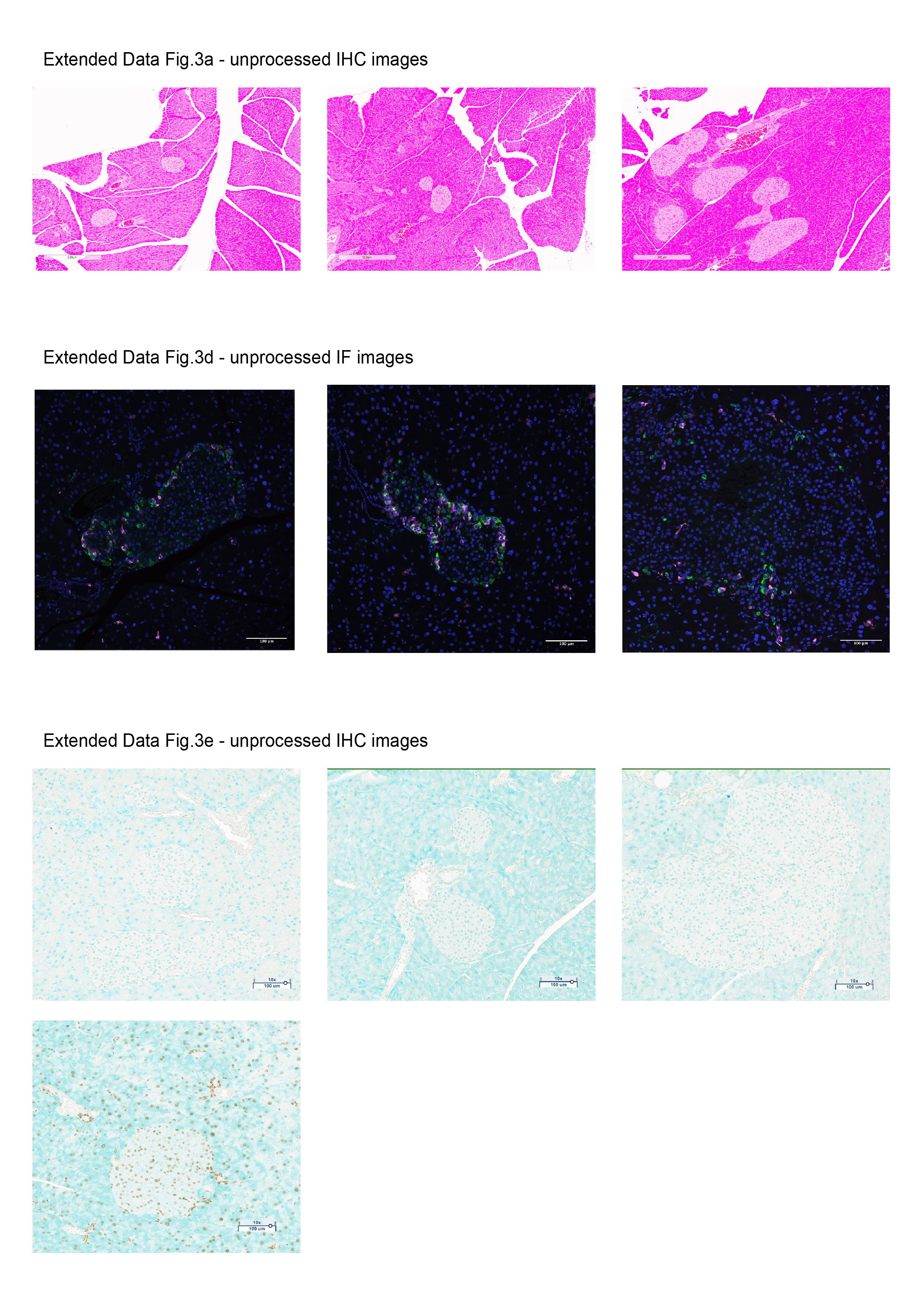

Supplement: Source Data Extended Data Fig. 3 — Unprocessed and uncropped images. [file 42255_2022_629_MOESM13_ESM.jpg]
